# Expanding indications for transcatheter aortic valve implantation: time for valve-in-valve in mechanical prosthesis? A systematic review and meta-analysis

**DOI:** 10.3389/fcvm.2026.1920900

**Published:** 2026-09-23

**Authors:** Julio Ruiz-Ruiz, Akash Jain, Mario García Gómez, Alexander Stepanenko, Filippo Pensotti, Marcelo Rodríguez, Carlos Real, Carlos Cortés Villar, Carlos Galán-Arriola, Blanca De Prada Martín, Manuel Carrasco Moraleja, Robert Willian Anastácio Alcântara, Alberto San Román, Borja Ibañez, Ignacio Amat-Santos

**Affiliations:** 1 Cardiology Department, University Clinic Hospital of Valladolid, Spain; 2 Centro Nacional de Investigación Cardiovascular (CNIC), Madrid, Spain; 3 Centro de Investigación Biomedica en Red (CIBERCV), Madrid, Spain; 4 Anesthesiology Department, University Clinic Hospital of Valladolid, Spain; 5 Clínica Aurus, Oliveira, Brazil; 6 Interventional Cardiology Unit, Hospital São Judas Tadeu, Oliveira, Brazil; 7 Cardiology Department, Fundación Jimenez Diaz, Madrid, Spain

**Keywords:** bleeding complications, mechanical aortic prosthesis, TAVI, transcatheter aortic valve implantation, valve-in valve

## Abstract

**Aims:**

Following the recent first-in-human experience of Valve in Mechanical Valve (ViMech), interest has emerged in understanding how frequently such an approach might be required in clinical practice. We performed an exploratory meta-analysis to characterize the long-term burden of valve-related complications and reintervention after mechanical aortic valve replacement.

**Methods:**

Systematic literature search identified studies reporting outcomes of the five most used mechanical aortic valve models (Medtronic/ATS, St. Jude, Carbomedics, Bicarbon, On-X). Descriptive analysis and random-effects meta-analysis assessed late all-cause and valve-related mortality and complications (bleeding, thromboembolism, valve thrombosis, endocarditis, paravalvular leak, reintervention), expressed per 100 patient-years.

**Results:**

Thirty-two studies encompassing 24,960 patients undergoing mechanical valve replacement were assessed. Isolated mechanical aortic valve replacement was reported in all 32 studies (13,890 patients; 98,987 patient-years; mean follow-up 7.1 years); mean age at implantation was 57.9 years (95% CI 56.3–59.6) and 43% (38–49) were female. Pooled all-cause mortality was 2.39 per 100 patient-years (95% CI 1.92–2.99) and valve-related mortality was 0.55 (0.41–0.73) per 100 patient-years. Bleeding (0.22 [0.16–0.30]) and thromboembolic events (0.18 [0.12–0.25]) were the leading causes of valve-related death. Thromboembolic events (0.94 [0.77–1.14]) and bleeding (0.85 [0.65–1.10]) were the most frequent non-fatal complications, considerably more common than reintervention (0.36 [0.28–0.48] per 100 patient-years). All-cause mortality and complication rates were broadly similar across valve models.

**Conclusions:**

Mechanical aortic valves show excellent long-term durability with low reintervention rates. Nevertheless, valve-related complications, mainly bleeding and thromboembolism, suggest a small but clinically relevant population in whom redo surgery may carry substantial risk, providing epidemiological context for emerging Valve in Mechanical Valve strategies.

**Systematic Review Registration:**

https://www.crd.york.ac.uk/PROSPERO/view/CRD420251002419, identifier CRD420251002419.

## Central Figure

**Figure:**
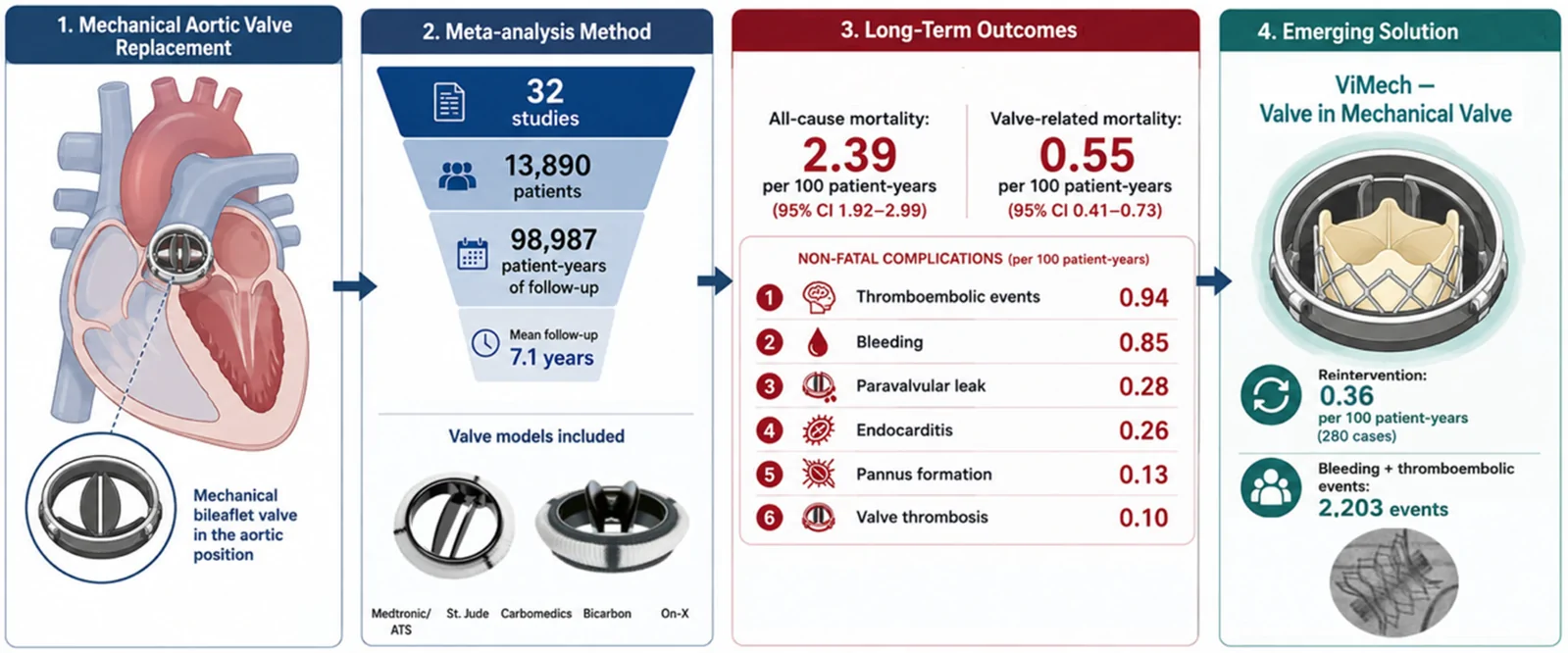

## Introduction

Valvular heart disease is a major cause of morbidity, mortality, and impaired quality of life worldwide (1, 2). Among these conditions, aortic valve disease represents one of the most prevalent and clinically relevant entities (3), with valve replacement constituting the standard treatment for patients requiring intervention (4, 5). This procedure can be performed using either mechanical or biological prostheses, depending on patient age, comorbidities, and life-expectancy. Mechanical valves are typically preferred in patients under 60 years of age with acceptable life expectancy and no formal contraindication to long-term anticoagulation (4, 5). Alternatively, biological prostheses, implanted either surgically (SAVR) or via transcatheter aortic valve implantation (TAVI), are selected based on individual risk profiles (4, 5).

Mechanical aortic valve prostheses have historically been regarded as the most durable option for surgical aortic valve replacement, particularly in younger patients ^(^6–8). Their long-term structural integrity has been consistently demonstrated across multiple decades of clinical experience. However, this durability comes at the cost of lifelong anticoagulation and its associated complications (9).

Until very recently, failure of a mechanical prosthesis was considered synonymous with the need for redo surgery, as transcatheter reintervention was thought to be infeasible (10). An isolated case report first described successful transcatheter valve-in-valve implantation into a failed mechanical aortic prosthesis facilitated by leaflet fracturing (11). More recently, our group reported the first structured, translational first-in-human study combining preclinical animal data with human feasibility of transcatheter aortic valve implantation within a mechanical aortic prosthesis safely stopping vitamin-k inhibitors afterwards, which has challenged this long-standing paradigm, opening a new conceptual space for transcatheter treatment in carefully selected high-risk patients (12).

Once its technical feasibility was demonstrated; a critical unanswered question is how frequently and in which scenario such an approach might be required in real-world practice. While mechanical structural valve failure is generally considered rare, even low event rates may translate into a clinically meaningful population when considered at scale, particularly among elderly or frail patients.

Accordingly, the present exploratory meta-analysis was undertaken to quantify the incidence of mechanical aortic valve–related events potentially leading to reintervention and to provide an epidemiological framework for emerging transcatheter solutions.

## Methods

### Literature search strategy and eligible criteria

This systematic review and meta-analysis were prospectively registered in PROSPERO on 1 March 2025 (CRD420251002419) and was conducted and reported in accordance with the PRISMA 2020 statement and the MOOSE (Meta-analyses Of Observational Studies in Epidemiology) guidelines; the completed PRISMA 2020 checklist is provided in Supplementary Table S1. No deviations from the registered protocol occurred during the conduct of this review.

A comprehensive literature search was performed in Lens.org, a scholarly aggregator that simultaneously indexes multiple bibliographic sources (PubMed/MEDLINE, Crossref, PubMed Central, CORE, OpenAlex and Lens Scholarly). The search combined the five prespecified mechanical aortic valve platforms with terms for the aortic position and a broad outcomes block, without language restriction; the final search was run in July 2026 (Figure 1). The complete query was: (“St Jude” OR “St. Jude” OR SJM OR “On-X” OR OnX OR Bicarbon OR CarboMedics OR “ATS valve” OR “ATS Medical” OR “Open Pivot” OR “Medtronic Open Pivot”) AND (“aortic valve replacement” OR “aortic position” OR aortic) AND (outcome* OR “long-term” OR “follow-up” OR experience OR results OR survival OR durability OR reoperation OR reintervention OR thrombosis OR thromboembolism OR bleeding OR endocarditis). Records were exported and de-duplicated (by DOI and normalised title); books, book chapters, dissertations, datasets, conference abstracts and preprints were removed as ineligible record types before screening. The search was complemented by backward and forward citation searching of the included studies and relevant reviews and by reference-list screening.

**Figure 1:**
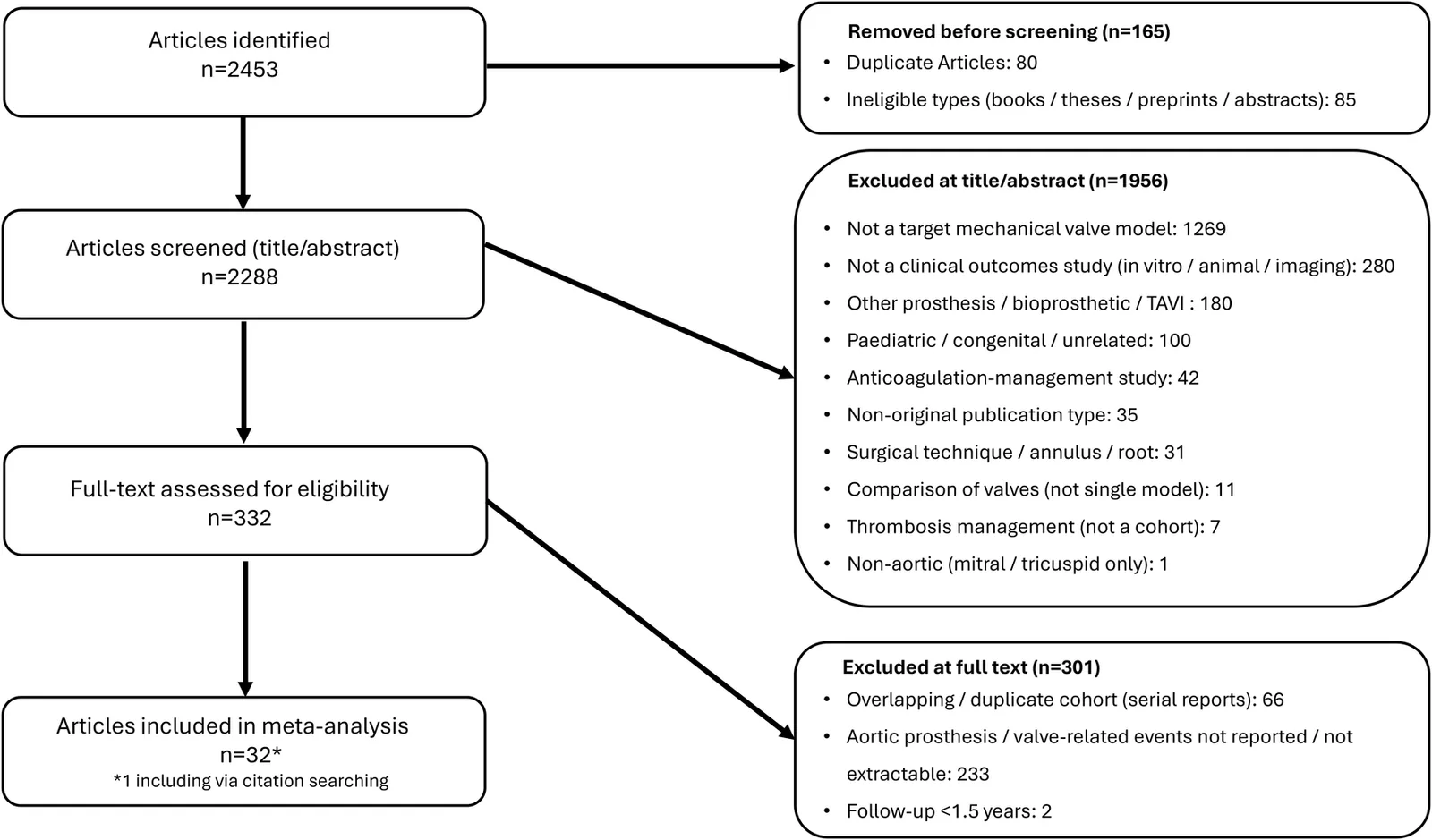
PRISMA article selection flow-chart.

We included observational cohorts (single- or multicentre, prospective or retrospective, including the observational arms of device registries and trials) reporting long-term outcomes of adults who underwent surgical implantation of one of the five prespecified contemporary bileaflet mechanical aortic valve platforms (Medtronic/ATS Open Pivot (13–16), St. Jude Medical (17–26),, On-X (27–34), Sorin Bicarbon (35–41), Carbomedics (42–49). Studies were eligible when they reported the number of prostheses implanted in the aortic position and at least one late valve-related outcome, with a mean follow-up of at least one year. Mixed-position cohorts were eligible, but clinical-event analyses were restricted *a priori* to the isolated aortic valve replacement (AVR) subgroup. We excluded studies that did not specify the number of aortic implants, evaluated exclusively non-aortic positions, reported neither valve-related mortality nor valve-related events, or had a mean follow-up shorter than one year; case reports, abstracts, preprints, reviews and other non-primary record types were not eligible. A study was not excluded solely because a single secondary outcome was unreported, which is why the number of contributing studies (k) varies across outcomes. The restriction to five platforms was prespecified as the most widely implanted contemporary bileaflet mechanical aortic prostheses with sufficient published long-term follow-up.

Possible overlap between cohorts was handled in two tiers. Reports with a high probability of overlap, four serially updated single-centre St. Jude Medical series describing progressively larger, largely overlapping populations, were excluded *a priori* from all analyses (primary set: 32 non-overlapping studies). Three reports judged at low-to-moderate risk of overlap were retained in the primary analysis and removed in a dedicated sensitivity analysis, keeping the most complete report of each same-device cluster (Supplementary Table S2).

#### Data extraction

Two reviewers independently screened titles/abstracts, assessed full texts and extracted study-level data (design, country, recruitment period, valve model and generation, number of aortic and total patients, age, sex, target INR, follow-up duration and completeness, cumulative person-time, outcome definitions and event counts) onto a standardised form; disagreements were resolved by consensus, and corresponding authors were contacted when required data were missing or ambiguous. The complete study-level characteristics of the included studies are provided in Supplementary Table S3.

#### Risk of bias

Methodological quality and risk of bias were assessed independently by two reviewers using the Newcastle–Ottawa Scale (NOS) adapted for single-arm surgical cohorts, omitting the Comparability domain and the selection of a non-exposed cohort, which require a comparison group, yielding a six-item instrument (three Selection and three Outcome items; maximum six stars), reporting domain-level judgements rather than a single summary score, with disagreements resolved by consensus. The complete domain-level assessment is provided in Supplementary Table S4.

### Statistical analysis

The primary estimand of this meta-analysis was the pooled incidence rate of valve-related mortality and morbidity among recipients of mechanical aortic valves, overall and stratified by valve model. Clinical-event analyses were restricted to patients undergoing isolated mechanical aortic valve replacement. Baseline characteristics were pooled across studies using random-effects models (logistic-normal for proportions and a random-effects mean for age) and are reported for the overall population and by valve model (Table 1). For each outcome, event rates were expressed per 100 patient-years to account for differences in follow-up duration and were calculated as the number of events divided by the total patient-years, multiplied by 100.

**Table 1:** Baseline characteristics and lesion and aetiology leading to replacement for each valve model.

Variable	Bicarbon *K* = 5	Carbomedics *K* = 7	Medtronic/ATS *K* = 4	On-X *K* = 7	St. Jude *K* = 9	Overall *K* = 32
Age, years	60.1 (58.5–61.6)	54.7 (49.8–59.7)	60.2 (57.9–62.5)	58.4 (55.4–61.3)	57.7 (53.7–61.7)	57.9 (56.3–59.6)
Female, %	43 (36–50)	44 (30–59)	42 (32–51)	41 (34–48)	47 (29–66)	43 (38–49)
Isolated AVR, %	54 (41–67)	89 (26–99)	62 (42–79)	57 (22–86)	91 (42–99)	76 (56–89)
MVR, %	37 (23–53)	8 (1–56)	32 (25–41)	38 (13–71)	6 (1–40)	18 (9–34)
AVR + MVR, %	11 (9–13)	2 (0–21)	9 (1–46)	1 (0–13)	3 (0–18)	3 (1–7)
CABG, %	14 (10–19)	4 (1–24)	21 (5–55)	13 (6–26)	14 (6–29)	12 (8–18)
First procedure, %	NE	55 (0–100)	89 (63–97)	92 (69–98)	90 (42–99)	88 (77–94)
Redo procedure, %	9.5	24 (0–97)	8 (1–53)	11 (6–21)	10 (1–58)	12 (7–19)
Stenosis, %	38 (0–100)	62 (36–83)	39 (3–93)	54 (10–93)	58 (13–93)	49 (33–66)
Regurgitation, %	1 (0–100)	19 (1–86)	44 (9–87)	17 (5–46)	28 (7–68)	22 (9–43)
Combined, %	1 (0–100)	12.1	11 (2–44)	19 (0–100)	15 (5–39)	12 (5–25)
Degenerative, %	47 (0–100)	31 (12–60)	NE	53 (24–80)	18 (0–91)	38 (20–59)
Rheumatic, %	24 (4–71)	39 (14–72)	NE	11 (2–47)	21 (5–59)	22 (13–35)
Congenital, %	5 (0–91)	6 (0–64)	NE	21 (2–82)	14 (6–28)	12 (6–23)
Endocarditis (etiology), %	4 (1–18)	7 (2–23)	5.1	5 (3–10)	7 (2–17)	6 (4–8)
Previous dysfunction, %	8 (3–18)	16 (4–45)	2 (2–2)	5 (2–15)	2 (0–11)	5 (3–9)
Other etiology, %	14 (0–100)	0.0	NE	2 (0–5)	2 (0–100)	3 (1–10)

AVR, Aortic Valve Replacement; MVR, Mitral Valve Replacement; TVR, Tricuspid Valve Replacement; CABG, Coronary Artery Bypass Graft; Proportions pooled with random-effects logistic-normal models (Hartung-Knapp CIs); age with a random-effects mean. Denominators: Proportions for procedure type (Age, sex, AVR, MVR, AVR + MVR, CABG, first/redo procedure) use the total study population as denominator; proportions for aortic lesion and aetiology use the number of isolated AVR patients. NE = not estimable. Estimates from a single contributing study (k = 1) are shown without a confidence interval.

Rather than relying on primary study-reported person-time, which was incompletely reported and often encompassed the overall surgical population rather than the isolated AVR subgroup, AVR-specific person-time was calculated as the product of the number of isolated AVR procedures and each study's mean follow-up. This estimate showed near-perfect agreement with the person-time reported by the studies that provided it and was robust to conservative reductions of the denominator; in addition, a sensitivity analysis incorporating apportioned whole-cohort person-time demonstrated no significant difference from the primary estimates. Nonetheless, because individual follow-up times may lack completeness or symmetry, these findings should be regarded as exploratory, as noted in the study limitations. Full results of this validation and sensitivity check are reported in Supplementary Table S5.

Pooled incidence rates were estimated with random-effects Poisson models on the log incidence-rate scale (IRLN). Between-study variance (*τ*^2^) was estimated by the empirical-Bayes (Paule–Mandel) method and confidence intervals were computed with the Hartung–Knapp adjustment; analyses were stratified by valve model. For every pooled outcome, and for each valve model and overall, we report the number of contributing studies (k), the number of eligible aortic patients (N), the number of events, the aortic patient-years, the pooled rate with 95% confidence interval, the heterogeneity (I² and *τ*²), and a 95% prediction interval. Heterogeneity was quantified with I² (>50% considered substantial).

Studies reporting no events for a given outcome were retained by applying a continuity correction of 0.5 events (the default add = 0.5 of the log incidence-rate transformation), so that a finite log-rate could be computed and the study still contributed to the pooled estimate. In sparse subgroups informed by very few studies the between-study variance becomes very large and the upper confidence or prediction limit is not reliably estimable; for readability, such upper limits exceeding 100 events per 100 patient-years are displayed as ∞ and the corresponding estimate is interpreted as unstable.

Univariable random-effects meta-regression on the log incidence-rate scale was the primary approach to explore heterogeneity; for each moderator (publication year, sample size, mean age and female proportion) we report the coefficient with 95% confidence interval, the moderator test (Q_M), the residual heterogeneity (R² and residual *τ*²) and the number of contributing studies. The absence of statistically significant associations was interpreted as inconclusive rather than as evidence that these variables do not explain heterogeneity. Univariable meta-regression for all-cause mortality and for reintervention is reported in Supplementary Table S6. Head-to-head differences between valve models were further explored with pairwise random-effects meta-regression on each pair of models; where the empirical-Bayes estimator did not converge, the DerSimonian–Laird estimator was used, and pairs with fewer than two studies per model were deemed not estimable (Supplementary Table S7).

Small-study effects were examined for outcomes informed by at least ten studies using contour-enhanced funnel plots and the Egger regression test, complemented by trim-and-fill, Baujat and leave-one-out diagnostics, acknowledging the limited validity of funnel-based methods for heterogeneous incidence-rate meta-analyses. Per-outcome heterogeneity and small-study-effect results are detailed in Supplementary Tables S8, S9, the corresponding contour-enhanced funnel plots in Supplementary Figure S1, and the leave-one-out sensitivity analysis for all-cause mortality in Supplementary Table S10; robustness to cohort overlap was additionally assessed in Supplementary Table S2. The certainty of evidence for the principal outcomes was appraised in a GRADE-informed manner (risk of bias, inconsistency, indirectness, imprecision and reporting bias) and rated low to very low.

Reporting followed PRISMA 2020 and MOOSE. A two-sided *P* < 0.05 was considered statistically significant, and meta-regression results were treated as exploratory. All statistical analyses were conducted using R software (version 4.2.0; R Foundation for Statistical Computing), employing the metafor, meta, forestplot, ggplot2 and dplyr packages.

### Ethics

This study is a systematic review and meta-analysis of previously published studies and did not require approval from an ethics committee.

## Results

### Sample and baseline characteristics

A total of 32 studies were included in the meta-analysis, encompassing 24,960 patients who underwent mechanical valve replacement, corresponding to 171,645 patient-years of cumulative follow-up (mean follow-up 6.9years). Of these, isolated mechanical aortic valve replacement was reported in all 32 studies, comprising 13,890 patients and 98,987 patient-years of follow-up (mean follow-up 7.1 years); the remaining patients underwent isolated mitral (7,878 patients; 24 studies), combined aortic-mitral (1,578 patients; 17 studies), or tricuspid (80 patients; 3 studies) valve replacement (Figure 2). Consistent with the pre-specified analysis plan, clinical-event analyses were restricted to the isolated aortic valve replacement subgroup. Baseline characteristics for the pooled cohort, stratified by valve model, are summarised in Table 1. Mean age at implantation was 57.9 (95% CI 56.3–59.6) years, and 43% (38–49) of patients were female.

**Figure 2:**
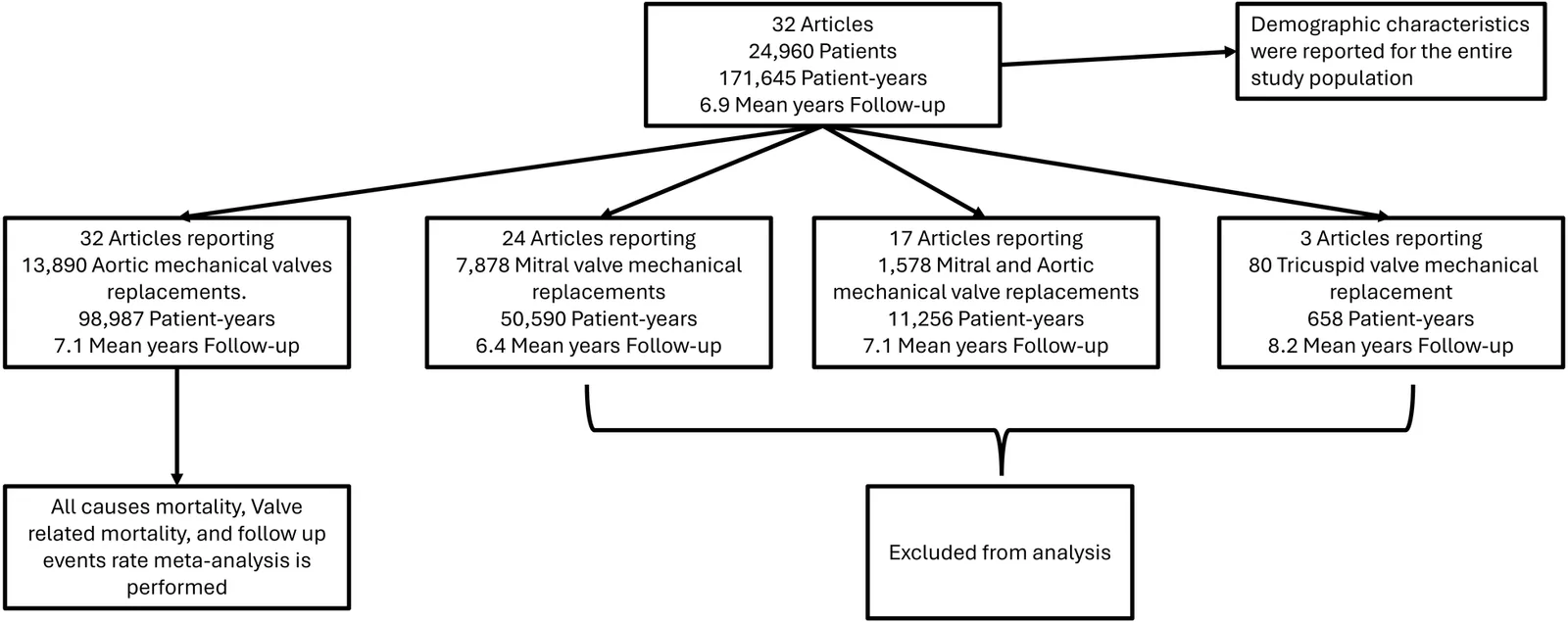
Meta-analysis patient selection scheme.

Across the pooled cohort, the study-level pooled proportion of isolated aortic valve replacement was 76% with combined aortic-mitral replacement in 3% and concomitant coronary artery bypass grafting being performed in 12% of patients. Most procedures were first-time interventions (88%; 95% CI 77–94), while 12% (95% CI 7–19) were redo procedures. Among patients undergoing aortic valve replacement, the underlying indication was isolated aortic stenosis in 49% (95% CI 33–66), pure aortic regurgitation in 22% (95% CI 9–43), and combined lesions in 12% (95% CI 5–25); degenerative disease was the most frequent aetiology, accounting for 38% (95% CI 20–59) of cases, followed by rheumatic (22%; 95% CI 13–35) and congenital (12%; 95% CI 6–23) aetiology.

Baseline characteristics stratified by valve model are summarised in Table 1. As shown in Figure 3, patient age at the time of surgery showed a downward trend across the study period (2003–2021), although this trend did not reach statistical significance (*β* = –0.348 years/year; *P* = 0.084; *k* = 24 studies).

**Figure 3:**
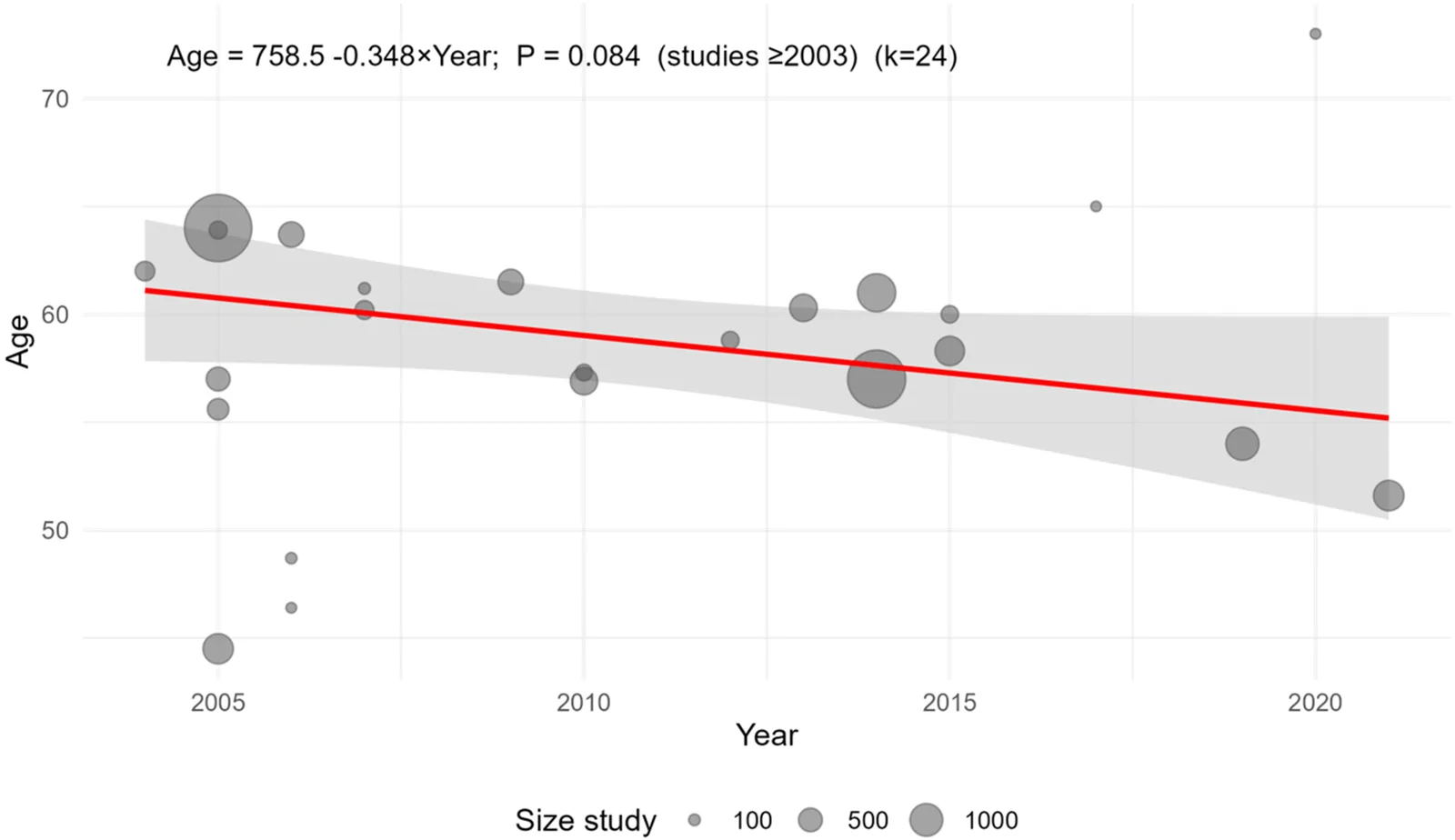
Adjusted by population scatter plot. Adjusted by population scatter plot showing mean age downslope tendency in patients treated with a mechanical valve replacement since 2003; the size-weighted regression. The downward trend did not reach statistical significance (*β* = –0.348 years/year; *P* = 0.084; *k* = 24 studies).

### Main outcomes and complications during follow up

Among patients undergoing isolated mechanical aortic valve replacement, the pooled all-cause mortality rate was 2.39 (95% CI 1.92–2.99) per 100 patient-years (*k* = 31 studies, 13,164 patients, 3,042 events, 94,631 patient-years), while valve-related mortality was 0.55 (95% CI 0.41–0.73) per 100 patient-years (*k* = 24, 11,806 patients, 533 events, 81,278 patient-years). The forest plot for all-cause mortality is presented in Figure 4.

**Figure 4:**
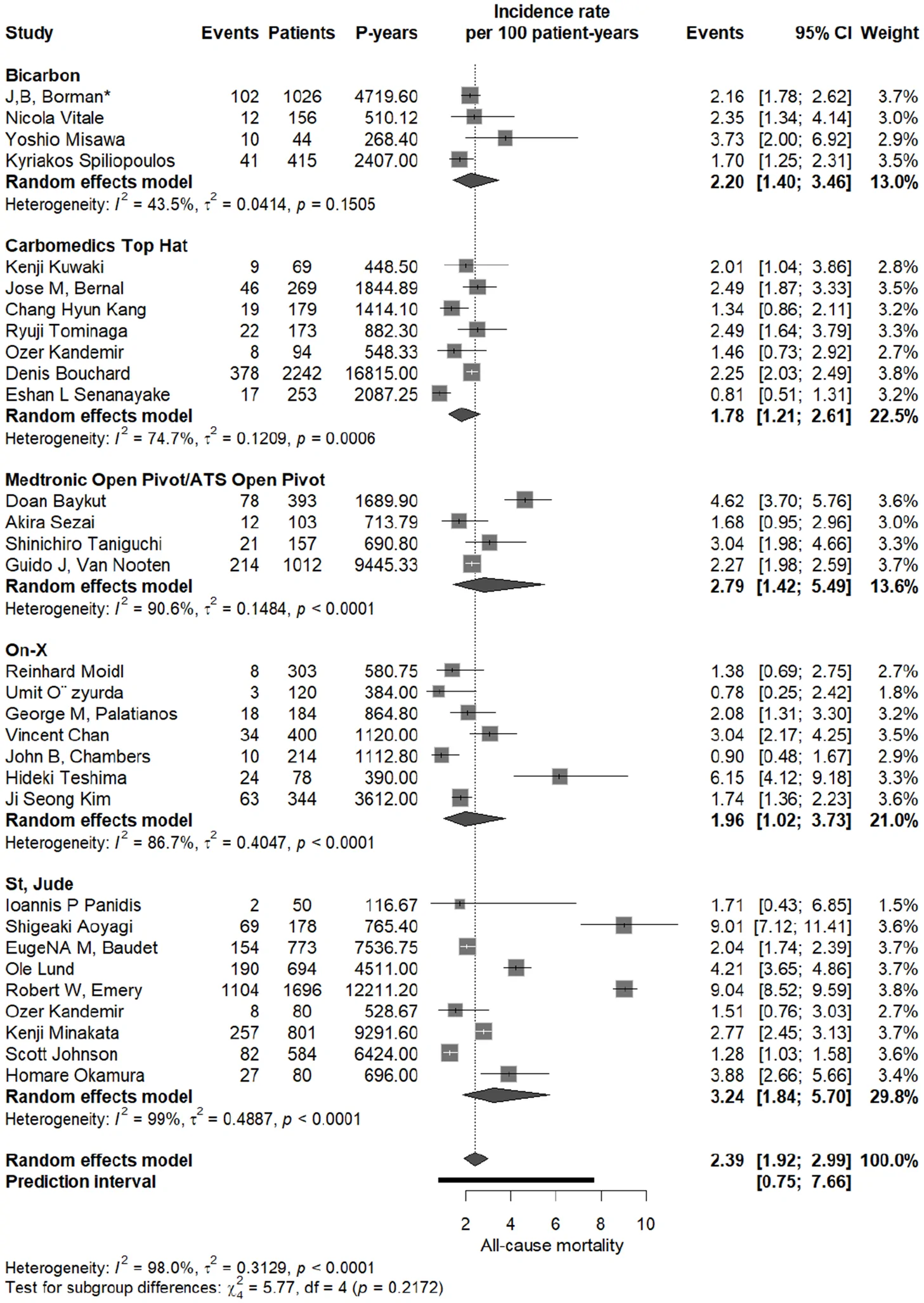
Forest plot for the all-cause mortality rate according to valve model, estimated with the random-effects model and shown with 95% prediction intervals.

The leading causes of valve-related death were bleeding and thromboembolism, with pooled incidence rates of 0.22 (0.16–0.30) and 0.18 (0.12–0.25) per 100 patient-years, respectively, followed by endocarditis (0.10 [0.07–0.14]), reintervention (0.07 [0.05–0.10]), valve thrombosis (0.04 [0.03–0.06]) and paravalvular leak (0.03 [0.02–0.06]). All-cause and valve-related mortality rates for the overall cohort and stratified by valve model are detailed in Table 2.

**Table 2:** All-cause mortality and valve-related cause of mortality for our cohort and each valve model expressed as rate per 100 patient-years.

Outcome	Valve	*k*	*N*	Events	PY	Rate	I2	PI
All-cause mortality	Bicarbon	4	1,641	165	7,905	2.20 (1.40–3.46)	55%	1.00–4.85
	Carbomedics	7	3,279	499	24,040	1.78 (1.21–2.61)	81%	0.70–4.53
	Medtronic/ATS	4	1,665	325	12,540	2.79 (1.42–5.49)	89%	0.69–11.32
	On-X	7	1,643	160	8,064	1.96 (1.02–3.73)	89%	0.36–10.55
	St. Jude	9	4,936	1,893	42,081	3.24 (1.84–5.70)	99%	0.59–17.89
	Overall	31	13,164	3,042	94,631	2.39 (1.92–2.99)	96%	0.75–7.66
Valve-related mortality	Bicarbon	5	2,367	68	12,261	0.57 (0.41–0.79)	0%	0.41–0.79
	Carbomedics	5	2,932	79	21,405	0.44 (0.23–0.82)	53%	0.14–1.35
	Medtronic/ATS	2	550	5	2,381	0.22 (0.01–4.53)	0%	0.01–4.53
	On-X	4	1,101	6	3,678	0.18 (0.12–0.29)	0%	0.12–0.29
	St. Jude	8	4,856	375	41,553	0.89 (0.54–1.46)	91%	0.24–3.31
	Overall	24	11,806	533	81,278	0.55 (0.41–0.73)	86%	0.17–1.75
- Thrombosis (death)	Bicarbon	3	1,908	1	9,586	0.03 (0.00–0.31)	0%	0.00–0.31
	Carbomedics	4	2,753	2	19,991	0.02 (0.00–0.11)	0%	0.00–0.11
	Medtronic/ATS	2	550	0	2,381	0.05 (0.00–13.60)	0%	0.00–13.60
	On-X	2	487	0	1,446	0.07 (0.01–0.89)	0%	0.01–0.89
	St. Jude	6	3,877	10	31,496	0.05 (0.02–0.12)	26%	0.01–0.21
	Overall	17	9,575	13	64,898	0.04 (0.03–0.06)	0%	0.03–0.06
- Thromboembolism (death)	Bicarbon	3	1,908	11	9,586	0.12 (0.06–0.25)	0%	0.06–0.25
	Carbomedics	4	2,753	18	19,991	0.10 (0.08–0.11)	0%	0.08–0.11
	Medtronic/ATS	2	550	2	2,381	0.13 (0.00–∞)	52%	0.00–∞
	On-X	2	487	2	1,446	0.19 (0.00–28.80)	0%	0.00–28.80
	St. Jude	6	3,877	94	31,496	0.24 (0.12–0.49)	73%	0.05–1.09
	Overall	17	9,575	127	64,898	0.18 (0.12–0.25)	47%	0.07–0.42
- Bleeding (death)	Bicarbon	4	2,323	16	11,993	0.14 (0.06–0.36)	14%	0.04–0.47
	Carbomedics	4	2,753	24	19,991	0.13 (0.07–0.23)	0%	0.07–0.23
	Medtronic/ATS	2	550	2	2,381	0.11 (0.01–1.31)	0%	0.01–1.31
	On-X	2	487	0	1,446	0.07 (0.01–0.89)	0%	0.01–0.89
	St. Jude	7	4,055	126	32,261	0.36 (0.23–0.55)	57%	0.15–0.85
	Overall	19	10,168	168	68,071	0.22 (0.16–0.30)	55%	0.09–0.53
- Endocarditis (death)	Bicarbon	4	2,323	7	11,993	0.06 (0.04–0.09)	0%	0.04–0.09
	Carbomedics	3	2,684	3	19,542	0.02 (0.00–0.23)	0%	0.00–0.23
	Medtronic/ATS	2	550	0	2,381	0.05 (0.00–13.60)	0%	0.00–13.60
	On-X	2	487	0	1,446	0.07 (0.01–0.89)	0%	0.01–0.89
	St. Jude	6	3,877	41	31,496	0.14 (0.10–0.19)	0%	0.10–0.19
	Overall	17	9,921	51	66,857	0.10 (0.07–0.14)	15%	0.05–0.18
- Paravalvular leak (death)	Bicarbon	2	882	0	4,866	0.03 (0.00–∞)	13%	0.00–∞
	Carbomedics	3	2,684	0	19,542	0.02 (0.00–0.75)	15%	0.00–1.63
	Medtronic/ATS	2	550	1	2,381	0.06 (0.02–0.21)	0%	0.02–0.21
	On-X	2	487	0	1,446	0.07 (0.01–0.89)	0%	0.01–0.89
	St. Jude	5	3,293	3	25,072	0.03 (0.00–0.18)	41%	0.00–0.72
	Overall	14	7,896	4	53,306	0.03 (0.02–0.06)	0%	0.02–0.06
- Reoperation (death)	Bicarbon	2	882	0	4,866	0.03 (0.00–∞)	13%	0.00–∞
	Carbomedics	3	2,684	15	19,542	0.08 (0.04–0.17)	0%	0.04–0.17
	Medtronic/ATS	NE						
	On-X	2	487	0	1,446	0.07 (0.01–0.89)	0%	0.01–0.89
	St. Jude	4	2,410	10	19,448	0.06 (0.03–0.14)	0%	0.03–0.14
	Overall	11	6,463	25	45,302	0.07 (0.05–0.10)	0%	0.05–0.10
- Others (death)	Bicarbon	4	2,323	26	11,993	0.19 (0.02–1.84)	87%	0.00–16.46
	Carbomedics	3	2,684	8	19,542	0.05 (0.00–28.56)	83%	0.00–∞
	Medtronic/ATS	NE						
	On-X	2	487	0	1,446	0.07 (0.01–0.89)	0%	0.01–0.89
	St. Jude	4	892	23	8,002	0.28 (0.01–6.52)	87%	0.00–∞
	Overall	13	6,386	57	40,983	0.15 (0.05–0.44)	89%	0.01–4.31

Rates are expressed per 100 patient-years on the isolated-aortic denominator, and each pooled estimate is reported with its number of contributing studies (k), aortic patients (N), events, aortic patient-years (PY), 95% confidence interval, I2, tau2 and a 95% prediction interval. Studies reporting no events for a given outcome contribute to the pooled estimate through a standard continuity correction (+0.5 events; the default of the log incidence-rate transformation). For readability, a confidence or prediction interval whose upper limit exceeds 100 per 100 patient-years is displayed as the infinity symbol. NE = not estimable (no contributing study). Per-outcome heterogeneity and small-study-effect details are reported in Supplementary Table S8.

Regarding late complications, thromboembolic events and bleeding were also the most frequent, with pooled rates of 0.94 (0.77–1.14) and 0.85 (0.65–1.10) per 100 patient-years, respectively, followed by paravalvular leak (0.28 [0.20–0.40]), endocarditis (0.26 [0.19–0.34]), pannus formation (0.13 [0.07–0.22]) and valve thrombosis (0.10 [0.07–0.13]). Valve reintervention for any cause occurred at a pooled rate of 0.36 (0.28–0.48) per 100 patient-years (*k* = 25, 11,069 patients, 259 events, 77,634 patient-years). Among reinterventions, the leading indications were endocarditis (0.13 [0.08–0.22]) and paravalvular leak (0.12 [0.09–0.16]), followed by pannus formation (0.06 [0.03–0.13]) and valve thrombosis (0.04 [0.03–0.07]).

### Differences across valve models

In unadjusted analyses, St. Jude and Medtronic/ATS Open Pivot valve models showed the numerically highest all-cause mortality rates, at 3.24 (95% CI 1.84–5.70) and 2.79 (1.42–5.49) per 100 patient-years, respectively, followed by Bicarbon (2.20 [1.40–3.46]), On-X (1.96 [1.02–3.73]) and Carbomedics (1.78 [1.21–2.61]) (Table 2). Between-model differences were not statistically significant. Univariable meta-regression did not identify publication year, sample size, or proportion of female patients as significant moderators of the pooled all-cause mortality rate (all *P* > 0.05), although mean age showed a significant positive association with the pooled mortality rate (*β* = 0.039 [95% CI 0.002–0.076]; *P* = 0.041; Figure 5).

**Figure 5:**
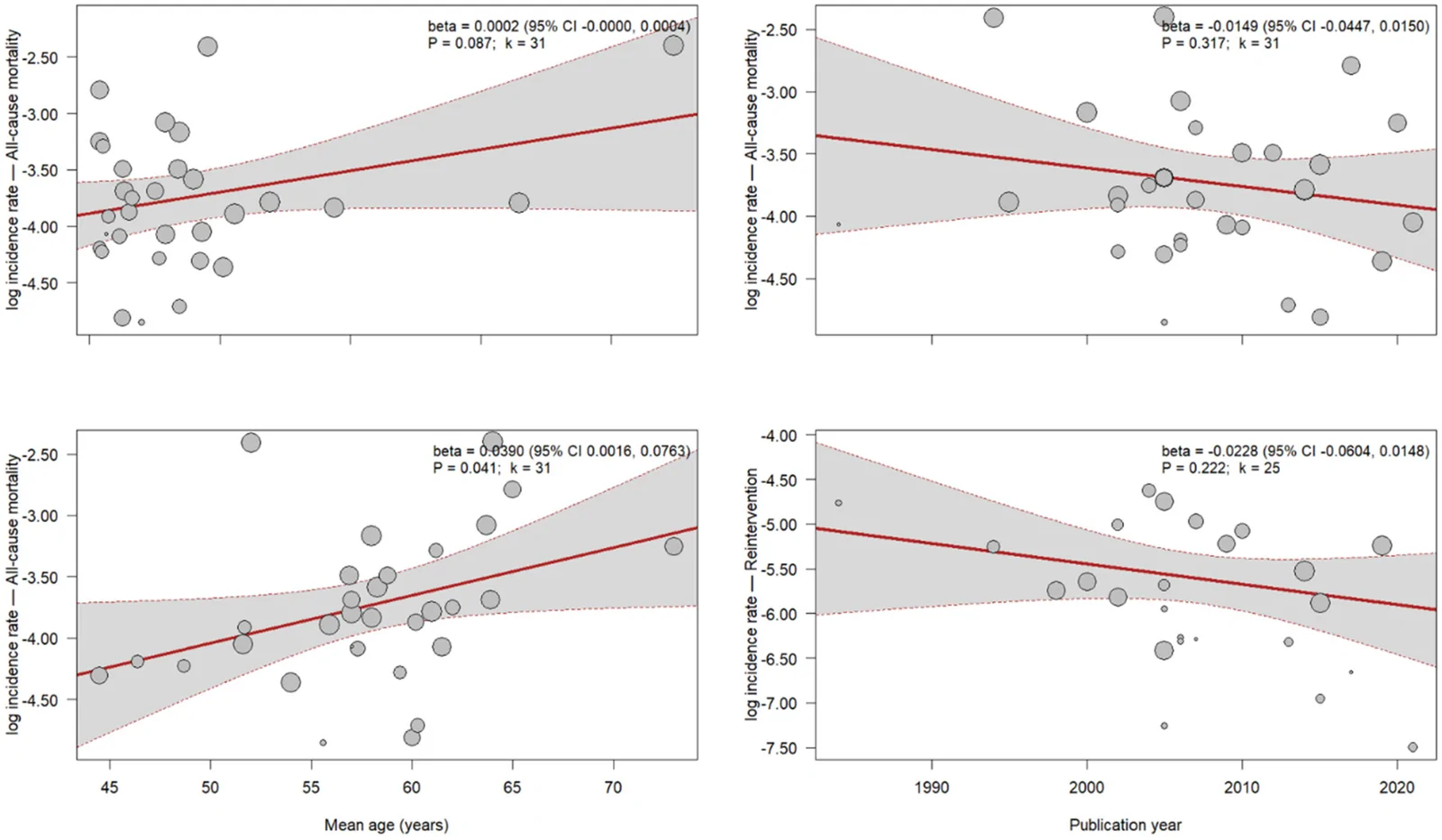
Meta-regression analyses evaluating the association between study-level characteristics and reported incidence rates.

Regarding valve-related complications, St. Jude valves showed the numerically highest unadjusted rates of valve-related mortality (0.89 [0.54–1.46]), valve thrombosis (0.13 [0.07–0.27]) and bleeding (1.17 [0.62–2.22]) and thromboembolic events (1.15 [0.74–1.80]). Among the remaining devices, Carbomedics valves showed the highest incidence of pannus formation (0.18 [0.02–1.49]), while Medtronic/ATS Open Pivot valves showed the highest rate of paravalvular leak (0.52 [0.02–18.01]). Confidence intervals for these complication rates overlapped broadly across valve models for most outcomes; however, pairwise random-effects meta-regression identified statistically significant differences in valve-related mortality between Bicarbon and On-X (rate ratio 0.32 [95% CI 0.15–0.68]; *P* = 0.009), between Medtronic/ATS and St. Jude (rate ratio 4.07 [1.03–16.13]; *P* = 0.047), and between On-X and St. Jude (rate ratio 4.99 [1.70–14.65]; *P* = 0.008) (Supplementary Table S7). Event rates for isolated mechanical aortic valve replacement, stratified by valve model, are presented in Table 3.

**Table 3:** Overall developed events during the follow-up and for each valve model expressed as rate per 100 patient-years.

Outcome	Valve	k	N	Events	PY	Rate	I2	PI
Thromboembolic events	Bicarbon	5	2,367	87	12,261	0.71 (0.42–1.21)	56%	0.27–1.90
	Carbomedics	6	3,026	125	21,953	0.87 (0.49–1.55)	77%	0.23–3.25
	Medtronic/ATS	3	653	27	3,094	0.75 (0.06–9.78)	84%	0.01–76.68
	On-X	7	1,643	66	8,064	0.88 (0.63–1.22)	11%	0.56–1.36
	St. Jude	9	4,936	726	42,081	1.15 (0.74–1.80)	93%	0.34–3.93
	Overall	30	12,625	1,031	87,454	0.94 (0.77–1.14)	84%	0.39–2.27
Valve thrombosis	Bicarbon	4	2,211	5	11,751	0.05 (0.02–0.15)	0%	0.02–0.15
	Carbomedics	5	2,757	8	20,108	0.05 (0.02–0.12)	0%	0.02–0.12
	Medtronic/ATS	4	1,665	5	12,540	0.05 (0.04–0.08)	0%	0.04–0.08
	On-X	5	1,165	1	6,554	0.07 (0.02–0.20)	0%	0.02–0.20
	St. Jude	9	4,936	50	42,081	0.13 (0.07–0.27)	61%	0.03–0.60
	Overall	27	12,734	69	93,035	0.10 (0.07–0.13)	22%	0.04–0.21
Pannus	Bicarbon	NE						
	Carbomedics	2	2,421	32	18,229	0.18 (0.02–1.49)	0%	0.02–1.49
	Medtronic/ATS	2	260	0	1,405	0.07 (0.06–0.09)	0%	0.06–0.09
	On-X	3	637	0	2,078	0.08 (0.02–0.30)	0%	0.02–0.30
	St. Jude	5	2,526	27	22,801	0.08 (0.02–0.39)	60%	0.00–1.59
	Overall	12	5,844	59	44,512	0.13 (0.07–0.22)	36%	0.05–0.36
Paravalvular leak	Bicarbon	5	2,367	21	12,261	0.20 (0.07–0.54)	54%	0.03–1.28
	Carbomedics	3	432	7	2,842	0.31 (0.02–5.54)	62%	0.00–36.44
	Medtronic/ATS	2	496	12	2,404	0.52 (0.02–18.01)	0%	0.02–18.01
	On-X	6	1,243	16	6,944	0.30 (0.13–0.70)	36%	0.07–1.30
	St. Jude	5	1,677	33	13,389	0.27 (0.11–0.64)	45%	0.06–1.14
	Overall	21	6,215	89	37,840	0.28 (0.20–0.40)	48%	0.10–0.81
Endocarditis	Bicarbon	5	2,367	34	12,261	0.30 (0.19–0.46)	0%	0.19–0.46
	Carbomedics	6	3,185	44	23,492	0.19 (0.09–0.42)	39%	0.05–0.75
	Medtronic/ATS	2	550	2	2,381	0.09 (0.00–27.20)	0%	0.00–27.20
	On-X	6	1,243	14	6,944	0.25 (0.13–0.47)	0%	0.13–0.47
	St. Jude	8	4,856	116	41,553	0.29 (0.14–0.61)	88%	0.04–1.99
	Overall	27	12,201	210	86,631	0.26 (0.19–0.34)	58%	0.10–0.65
Bleeding	Bicarbon	5	2,367	90	12,261	0.77 (0.45–1.31)	59%	0.28–2.08
	Carbomedics	6	3,026	136	21,953	0.77 (0.45–1.31)	69%	0.25–2.35
	Medtronic/ATS	3	653	14	3,094	0.49 (0.12–2.10)	35%	0.06–3.92
	On-X	7	1,643	54	8,064	0.72 (0.35–1.49)	74%	0.13–3.96
	St. Jude	9	4,936	878	42,081	1.17 (0.62–2.22)	98%	0.19–7.19
	Overall	30	12,625	1,172	87,454	0.85 (0.65–1.10)	92%	0.25–2.86
Reintervention (total)	Bicarbon	5	2,367	46	12,261	0.43 (0.23–0.79)	49%	0.14–1.30
	Carbomedics	7	3,279	93	24,040	0.35 (0.15–0.78)	76%	0.06–2.17
	Medtronic/ATS	NE						
	On-X	6	1,340	18	7,484	0.29 (0.10–0.83)	60%	0.03–2.54
	St. Jude	7	4,083	102	33,849	0.33 (0.21–0.53)	62%	0.12–0.89
	Overall	25	11,069	259	77,634	0.36 (0.28–0.48)	68%	0.13–1.01
- reint: PVL	Bicarbon	1	156	2	510	0.39 (0.10–1.57)	-	-
	Carbomedics	5	3,006	30	22,078	0.15 (0.10–0.21)	0%	0.10–0.21
	Medtronic/ATS	NE						
	On-X	3	565	3	1,836	0.20 (0.10–0.38)	0%	0.10–0.38
	St. Jude	6	4,033	27	33,732	0.09 (0.06–0.13)	0%	0.06–0.13
	Overall	15	7,760	62	58,155	0.12 (0.09–0.16)	0%	0.09–0.16
- reint: Endocarditis	Bicarbon	1	156	3	510	0.59 (0.19–1.82)	–	–
	Carbomedics	4	2,833	15	21,196	0.08 (0.04–0.15)	0%	0.04–0.15
	Medtronic/ATS	1	393	1	1,690	0.06 (0.01–0.42)	–	–
	On-X	4	909	6	5,448	0.18 (0.03–1.16)	48%	0.01–4.17
	St. Jude	6	4,033	40	33,732	0.12 (0.05–0.29)	68%	0.02–0.75
	Overall	16	8,324	65	62,575	0.13 (0.08–0.22)	60%	0.03–0.55
- reint: Thrombosis	Bicarbon	1	156	0	510	0.10 (0.01–1.57)	-	-
	Carbomedics	4	2,833	8	21,196	0.05 (0.03–0.08)	0%	0.03–0.08
	Medtronic/ATS	NE						
	On-X	3	565	1	1,836	0.11 (0.07–0.17)	0%	0.07–0.17
	St. Jude	5	3,953	6	33,203	0.03 (0.01–0.10)	19%	0.00–0.18
	Overall	13	7,507	15	56,745	0.04 (0.03–0.07)	0%	0.03–0.07
- reint: Pannus	Bicarbon	1	156	0	510	0.10 (0.01–1.57)	-	-
	Carbomedics	4	2,833	33	21,196	0.15 (0.05–0.42)	15%	0.03–0.66
	Medtronic/ATS	NE						
	On-X	2	381	0	971	0.11 (0.01–1.32)	0%	0.01–1.32
	St. Jude	5	3,953	3	33,203	0.02 (0.00–0.09)	20%	0.00–0.18
	Overall	12	7,323	36	55,880	0.06 (0.03–0.13)	43%	0.01–0.36
- reint: Others	Bicarbon	1	156	0	510	0.10 (0.01–1.57)	–	–
	Carbomedics	4	685	2	4,929	0.08 (0.02–0.31)	0%	0.02–0.31
	Medtronic/ATS	NE						
	On-X	2	422	1	4,002	0.05 (0.00–∞)	0%	0.00–∞
	St. Jude	6	4,726	31	40,740	0.07 (0.02–0.24)	77%	0.01–0.99
	Overall	13	5,989	34	50,181	0.09 (0.05–0.15)	25%	0.03–0.24

Rates are expressed per 100 patient-years on the isolated-aortic denominator, and each pooled estimate is reported with its number of contributing studies (k), aortic patients (N), events, aortic patient-years, 95% confidence interval, I2, tau2 and a 95% prediction interval. Cause-specific reinterventions are reported separately and are not additive components of the overall reintervention rate. Studies reporting no events for a given outcome contribute to the pooled estimate through a standard continuity correction (+0.5 events; the default of the log incidence-rate transformation). For readability, a confidence or prediction interval whose upper limit exceeds 100 per 100 patient-years is displayed as the infinity symbol. NE = not estimable (no contributing study). Per-outcome heterogeneity and small-study-effect details are reported in Supplementary Table S9.

## Discussion

The main findings of the present exploratory meta-analysis can be summarised as follows: I) Mechanical aortic valve prostheses demonstrate excellent long-term durability with low rates of valve-related mortality and reintervention but the use of mechanical prostheses has progressively declined over the last two decades; II) among the leading commercially available valve models, no significant differences were observed in unadjusted all-cause mortality during long-term follow-up, although an exploratory pairwise analysis suggested higher valve-related mortality in specific models; III) despite the very low incidence of intrinsic structural valve degeneration, the majority of adverse events and valve-related deaths were associated with anticoagulation-related complications; IV) pannus formation, infective endocarditis, and paravalvular leak were comparatively uncommon; and V) although valve reintervention occurred at a low incidence rate, it was not negligible when examined across large patient-year exposure.

The recent report demonstrating the feasibility of TAVI within bileaflet mechanical aortic valves has opened a novel therapeutic perspective for patients presenting with mechanical valve dysfunction or major valve-related complications and may ultimately influence future valve selection paradigms in SAVR. Current mechanical valve designs have evolved substantially from earlier cage-ball and monodisc prostheses toward bileaflet tilting-disk platforms, achieving improved haemodynamic performance and long-term durability (12). However, the commercially available models have not been extensively compared, and differences in long-term outcomes across valve platforms remain incompletely understood. Consequently, the real-world need for eventual Valve in Mechanical Valve interventions in the context of contemporary mechanical valve technology has remained largely undefined.

In our analysis, mechanical prostheses in the aortic position showed excellent long-term performance, with low rates of all-cause mortality and valve-related mortality. No significant differences in all-cause mortality were identified across valve models; however, an exploratory pairwise analysis suggested higher valve-related mortality for St. Jude valves compared with On-X and Medtronic/ATS Open Pivot valves. Given the number of pairwise comparisons performed, the observational design of the included studies, and the limited number of contributing studies in some subgroups, this finding should be interpreted as hypothesis-generating.

Regardless of mechanical valves' long-term performance, they are used less often in patients undergoing surgical aortic valve replacement. This is mainly due to advances in transcatheter valve-in-valve techniques for failed bioprosthetic valves, combined with the increasing availability of less invasive treatment options (12). A large registry including more than 100,000 patients undergoing aortic valve replacement demonstrated a decline in mechanical valve utilisation over time despite evidence of lower risk-adjusted mortality in patients ≤60 years receiving mechanical valves (51) and the particular greatest survival advantage in younger patients under 50 years of age. This benefit may extend up to 55–60 years (6–8, 52–55). However, that large cohort did not differentiate outcomes by valve model or valve-related events and likely included substantial overlap with studies incorporated in the present meta-analysis, precluding direct inclusion in our dataset. From a lifetime-management perspective, this durability advantage is clinically relevant: younger patients who select a bioprosthetic valve because of its perceived transcatheter valve-in-valve treatability face a high probability of eventually requiring reintervention, whereas mechanical prostheses rarely fail structurally and, when they do, may in carefully selected cases become suitable of a transcatheter ViMech approach.

In addition, the requirement for lifelong anticoagulation remains a fundamental limitation of mechanical valve therapy. The continued reliance on vitamin K antagonists, reinforced by the unfavourable outcomes of prospective randomised trials evaluating direct oral anticoagulants in patients with mechanical valves (50), contributes to recurrent haemorrhagic and thromboembolic complications with significant implications for quality of life and long-term prognosis. In our analysis, bleeding and thromboembolic events accounted for the majority of valve-related deaths and non-fatal complications, underscoring the central role of anticoagulation, rather than structural failure, in determining outcomes after mechanical aortic implantation.

Other potential complications are mechanical and structural valve dysfunction and the need for a surgical redo valve replacement. In our analysis, these mechanical and structural complications were infrequent.

The need for redo valve replacement was infrequent (0.36 per 100 patient-years) and encompassed a broad range of aetiologies, most commonly infective endocarditis and paravalvular leak, followed by refractory prosthetic thrombosis with haemodynamic compromise, obstructive pannus and irreversible leaflet dysfunction.

Not every case within these categories should be regarded as a potential ViMech indication. Most episodes of infective endocarditis still require surgical explantation;. Similarly, paravalvular leak are unsuitable candidates for ViMech and interventional leak occlusion should be considered. Given this heterogeneity, each of these conditions should be evaluated individually rather than treated as an undifferentiated pool of potential ViMech candidates. From a population-level perspective, although the pooled incidence of valve reintervention was low, extrapolating across the 77,634 patient-years accumulated by the 25 studies reporting this outcome corresponds to approximately 280 reinterventions in the analysed cohorts. More interesting is the potential indication in case of bleeding and thromboembolic events since they are considerably more frequent, together accounting for 2,203 reported events across 87,454 patient-years, and define a substantially larger population living with mechanical valve-related morbidity. How many of these events would ultimately translate into a procedure is not clear, since not every bleeding or thromboembolic episode reflects irreversible prosthesis dysfunction, and no empirical basis currently exists to estimate what proportion of them would require valve replacement. Under conservative assumptions that 5%, 10%, or 20% of these episodes ultimately prompt valve reintervention, the estimated number of patients potentially requiring valve replacement increases to approximately 390, 500, and 720 cases, respectively. While these figures are extrapolative and hypothesis-generating, what this burden does represent, largely driven by anticoagulation-related complications rather than structural failure, is the epidemiological motivation for continued development of Valve in Mechanical Valve strategies, even though the true size of the population that will ultimately benefit from them remains to be defined.

In this setting, the emergence of Valve in Mechanical Valve (ViMech) represents a promising adjunctive strategy for carefully selected patients who develop valve-related complications but are unsuitable candidates for redo surgery. Early preclinical studies and compassionate-use reports in humans have demonstrated the technical feasibility and short-term safety of this approach, supporting its potential role in real-world high-risk scenarios (11). Expanding TAVI indications into the mechanical setting does not aim to replace primary surgical mechanical valve replacement. Instead, it may serve, in carefully selected cases, as a potential transcatheter bailout strategy for patients who develop valve-related complications and are unsuitable candidates for redo surgery.

Important uncertainties remain. The sewing ring and residual native leaflet material are left *in situ*, and the long-term thrombogenicity of the resulting device-prosthesis interface is not known; there is currently no evidence that vitamin K antagonist therapy can be safely discontinued after a ViMech procedure. Preliminary data from our own group's preclinical and first-in-human experience (12) offer some early reassurance on this point. In the ten pigs that underwent the procedure, anticoagulation was changed to aspirin 100 mg daily without thrombotic or bleeding complications at 1–2 months of follow-up. Among the three patients described in that same first-in-human study, anticoagulation was similarly changed to aspirin in the first two patients and to apixaban in the third because of persistent atrial fibrillation. At 2-year follow up, all three patients remain alive and asymptomatic, with normally functioning devices; control CT demonstrated no leaflet thrombosis, and no further bleeding or ischaemic events were recorded. These findings, drawn from a very small and short-term experience, provide an early signal that antithrombotic de-escalation may be feasible after ViMech.

Coronary access, the risk of annular injury, new conduction disturbances, and procedural feasibility across the range of mechanical valve designs also remain to be characterised. The procedure is technically demanding and may require emergency surgical conversion, a risk that is not negligible even in conventional TAVI (56); cerebral embolisation is a further concern specific to this setting, since manipulation or fracture of mechanical leaflets, pannus, or thrombus could generate an embolic burden different from that of native-valve TAVI, and whether cerebral embolic protection has a role in early feasibility procedures is unresolved (57).

### Several limitations must be acknowledged

The exploratory nature of this meta-analysis and the heterogeneity of the included studies, particularly with regard to follow-up duration and event reporting. Patient-years were estimated using reported follow-up rather than individual-level data (validated against reported person-time in Supplementary Table S5), and baseline characteristics reflect overall study populations rather than isolated aortic valve replacement cohorts specifically. Incomplete reporting of certain complications limited the number of studies available for specific outcome analyses, as reflected in the variable k across outcomes in Tables 2, 3. Finally, the absence of patient-level anticoagulation data precluded assessment of the adequacy of anticoagulation at the time of adverse events.

## Conclusions

Mechanical aortic valve prostheses demonstrate excellent long-term durability, with low, model-independent rates of structural failure and reintervention. Most valve-related deaths and non-fatal complications are instead driven by anticoagulation-related bleeding and thromboembolism rather than prosthesis degeneration. These data define, for the first time, the epidemiological scale of complications that could plausibly be addressed by a Valve in Mechanical Valve approach, providing a rational, quantitative foundation for its development as a transcatheter alternative to redo surgery. Patients most likely to benefit include those undergoing structural valve reintervention, together with a still-undefined subset of the larger bleeding/thromboembolic population, since candidacy ultimately depends on individualising the underlying aetiology rather than the complication category as a whole.

Standardised Valve in Mechanical Valve approaches may help bridge the gap between the long-term durability of mechanical prostheses and the reintervention flexibility traditionally associated with bioprosthetic valves; if validated, this could favourably inform the lifetime-management calculus for younger patients, although this remains a hypothesis-generating consideration that would require prospective confirmation before it can influence valve selection in clinical practice.

## Data Availability

The original contributions presented in the study are included in the article/Supplementary Material, further inquiries can be directed to the corresponding author/s.
