# Supplementary material for: Expanding indications for transcatheter aortic valve implantation: time for valve-in-valve in mechanical prosthesis? A systematic review and meta-analysis
Source: Front Cardiovasc Med. 2026 Sep 23;13:1920900. doi: 10.3389/fcvm.2026.1920900 (PMC13643693; doi:10.3389/fcvm.2026.1920900)
Supplement: Supplementary file 1 [file Supplementaryfile1.docx]

**Expanding Indications for Transcatheter Aortic Valve Implantation: Time for valve-in-valve in mechanical prosthesis? A Systematic Review and Meta-Analysis**

Ruiz-Ruiz et al.

**SUPPLEMENTARY MATERIAL**

**Supplementary Table 1. PRISMA 2020 checklist.**

| Section and Topic | Item # | Checklist item | Location where item is reported |
| --- | --- | --- | --- |
| TITLE | | | |
| Title | 1 | Identify the report as a systematic review. | Title page: identified as "A Systematic Review and Meta-Analysis". |
| ABSTRACT | | | |
| Abstract | 2 | See the PRISMA 2020 for Abstracts checklist. | Abstract: structured (Aims, Methods, Results, Conclusions). |
| INTRODUCTION | | | |
| Rationale | 3 | Rationale for the review in the context of existing knowledge. | Introduction. |
| Objectives | 4 | Explicit statement of objective(s) or question(s). | Introduction (final paragraph); primary estimand: pooled incidence rate of mechanical aortic valve-related events after isolated aortic valve replacement, overall and by valve model. |
| METHODS | | | |
| Eligibility criteria | 5 | Inclusion/exclusion criteria and how studies were grouped for the syntheses. | Methods, Literature search and eligibility: five prespecified mechanical valve models; an outcome measured but not reported was treated as missing rather than as grounds for exclusion; reports excluded if the aortic-position number was not specified, if exclusively mitral/pulmonary/tricuspid, or if mean follow-up <1.5 years; syntheses grouped by valve model. |
| Information sources | 6 | All databases, registers, sources searched, and the date last searched. | Methods, Literature search: primary search in Lens.org (which simultaneously indexes PubMed/MEDLINE, Crossref, PubMed Central, CORE, OpenAlex and Lens Scholarly), complemented by backward and forward citation searching and reference-list screening; final search July 2026. Embase and Scopus were not searched directly. |
| Search strategy | 7 | Full search strategies for all databases/registers, including filters and limits. | Methods, Literature search; complete Boolean query provided in Supplementary Material (no language restriction; ineligible record types removed before screening). |
| Selection process | 8 | Methods to decide eligibility; number of reviewers; independence; automation. | Methods: two reviewers independently screened titles/abstracts and full texts; disagreements resolved by consensus. |
| Data collection process | 9 | Methods to collect data; reviewers; independence; author contact; automation. | Methods: two reviewers independently extracted data using a piloted form; disagreements resolved by consensus; outcomes not reported were recorded as missing. |
| Data items | 10a | All outcomes for which data were sought. | Methods, Statistical analysis; Results, Main outcomes: all-cause mortality; valve-related mortality (thrombosis, thromboembolism, bleeding, endocarditis, paravalvular leak); reintervention and its causes; thromboembolic events, valve thrombosis, pannus, paravalvular leak, endocarditis, bleeding, structural degeneration - all per 100 patient-years. |
|  | 10b | Other variables sought; assumptions about missing/unclear information. | Results, Baseline characteristics and Table 1 (age, sex, valve position, lesion/aetiology, redo status, CABG). Assumptions for missing data (person-time reconstruction; unreported outcomes as missing) are stated in Methods. |
| Study risk of bias assessment | 11 | Tool(s); number of reviewers; independence; automation. | Methods: Newcastle-Ottawa Scale adapted for single-arm surgical cohorts; two reviewers independently, disagreements resolved by consensus; domain-level judgements in Supplementary Material. |
| Effect measures | 12 | Effect measure(s) for each outcome. | Methods, Statistical analysis: incidence rate per 100 patient-years (log incidence-rate scale) with 95% confidence interval. |
| Synthesis methods | 13a | Deciding which studies were eligible for each synthesis. | Methods: analyses stratified by valve model and overall. |
|  | 13b | Preparing the data for synthesis (missing statistics, conversions). | Methods: aortic-specific person-time = number of isolated aortic prostheses x mean follow-up; zero-event studies retained with a 0.5 continuity correction. |
|  | 13c | Methods to tabulate or visually display results. | Tables 1-3; Figures 3-5. |
|  | 13d | Synthesis model(s), heterogeneity methods, and software. | Methods: random-effects Poisson model on the log incidence-rate scale; between-study variance by empirical-Bayes (Paule-Mandel); Hartung-Knapp confidence intervals; I2, tau2 and 95% prediction intervals; R (metafor, meta packages). |
|  | 13e | Methods to explore causes of heterogeneity. | Methods: univariable random-effects meta-regression (publication year, sample size, mean age, female proportion); Figure 5. |
|  | 13f | Sensitivity analyses. | Methods: leave-one-out; overlap sensitivity (all studies vs de-duplicated set); person-time method (estimated vs reported-apportioned) and validation against reported person-time; Supplementary Material. |
| Reporting bias assessment | 14 | Methods to assess risk of bias due to missing results. | Methods: contour-enhanced funnel plots and Egger's regression test for outcomes with >=10 studies, complemented by trim-and-fill, Baujat and leave-one-out; Supplementary Material. |
| Certainty assessment | 15 | Methods to assess certainty in the body of evidence. | Methods: GRADE-informed appraisal (risk of bias, inconsistency, indirectness, imprecision, reporting bias). |
| RESULTS | | | |
| Study selection | 16a | Results of the search and selection; flow diagram. | Results / Figure 1 (PRISMA 2020 flow diagram): 2,453 records identified; 2,288 screened; 1,956 excluded at title/abstract; 332 assessed in full text; 32 studies included. One study not indexed in Lens (Ozyurda 2005) added through citation searching. |
|  | 16b | Studies excluded at full text, with reasons. | Results; categorical exclusion reasons with counts; candidate/excluded list in Supplementary Material. |
| Study characteristics | 17 | Cite each included study and present its characteristics. | Results, Baseline characteristics; Table 1; included studies grouped by valve model in the References. |
| Risk of bias in studies | 18 | Per-study risk-of-bias assessments. | Supplementary Material (Newcastle-Ottawa domain-level table). |
| Results of individual studies | 19 | Per-study summary statistics and effect estimates. | Figure 4 (forest plot: per-study rates with 95% CI, events and patient-years); Table 1. |
| Results of syntheses | 20a | Characteristics and risk of bias among contributing studies. | Results; Table 1; overall risk of bias summarised in the Results. |
|  | 20b | All statistical syntheses; summary estimate, precision, heterogeneity. | Results; Tables 2-3 (k, N, events, patient-years, rate with 95% CI, I2, tau2, 95% prediction interval); Figure 4. |
|  | 20c | Results of investigations of heterogeneity. | Results, Differences across valve models; Figure 5. |
|  | 20d | Results of sensitivity analyses. | Results / Supplementary Material: leave-one-out and person-time sensitivity and validation. |
| Reporting biases | 21 | Assessments of risk of bias due to missing results per synthesis. | Results / Supplementary Material: funnel plots, Egger's test and trim-and-fill for the principal outcomes. |
| Certainty of evidence | 22 | Certainty in the body of evidence per outcome. | Results / Discussion: GRADE-informed certainty for the principal outcomes. |
| DISCUSSION | | | |
| Discussion | 23a | General interpretation in the context of other evidence. | Discussion. |
|  | 23b | Limitations of the evidence included. | Discussion, Limitations: exploratory nature; between-study heterogeneity; person-time estimated as patients x mean follow-up; baseline characteristics reflecting overall (not isolated-AVR) populations; suspected overlapping cohorts (addressed by the overlap sensitivity analysis). |
|  | 23c | Limitations of the review processes. | Discussion, Limitations: cohort-overlap handling and databases not searched directly (Embase/Scopus). |
|  | 23d | Implications for practice, policy and future research. | Discussion; Conclusions. |
| OTHER INFORMATION | | | |
| Registration and protocol | 24a | Registration information (register name and number). | Methods: prospectively registered in PROSPERO (CRD420251002419). |
|  | 24b | Where the protocol can be accessed. | Methods: PROSPERO record CRD420251002419. |
|  | 24c | Amendments to registration or protocol. | Methods: no deviations from the registered protocol. |
| Support | 25 | Sources of support and role of funders. | Title page/Funding: Instituto de Salud Carlos III (PI24/00388) and Government of Castilla y Leon (GRS 2917/A1/2023); the funders had no role in the design, analysis or reporting of the review. |
| Competing interests | 26 | Competing interests of authors. | Title page: declared. |
| Availability of data, code and other materials | 27 | What is publicly available and where. | Data availability statement: study-level data and the R analysis code are available from the corresponding author on reasonable request / in Supplementary Material. |

From: Page MJ, et al. The PRISMA 2020 statement. BMJ 2021;372:n71. Licensed under CC BY 4.0.

## **Supplementary Table 2. Sensitivity analysis removed 3 overlap studies with low-middle risk overlapping.**

| **Outcome (per 100 patient-years)** | **Full set including 4 high-overlap St. Jude cohorts**  **(36 studies)** | **Primary analysis**  **(32 studies)** | **Low-to-moderate-overlap reports removed**  **(29 studies)** |
| --- | --- | --- | --- |
| **All-cause mortality** | 2.59 (2.10-3.20) | 2.39 (1.92-2.99) | 2.44 (1.93-3.09) |
| **Valve-related mortality** | 0.60 (0.45-0.78) | 0.55 (0.41-0.73) | 0.56 (0.41-0.77) |
| **Reintervention** | 0.41 (0.31-0.54) | 0.36 (0.28-0.48) | 0.35 (0.26-0.48) |
| **Bleeding** | 0.96 (0.74-1.25) | 0.85 (0.65-1.10) | 0.84 (0.63-1.12) |
| **Thromboembolic events** | 1.02 (0.85-1.24) | 0.94 (0.77-1.14) | 0.96 (0.78-1.19) |

Values are pooled incidence rates per 100 patient-years (95% CI), random-effects Poisson model on the log incidence-rate scale (Empirical Bayes/Paule-Mandel τ², Hartung-Knapp CIs).

**Supplementary Table 3. Study-level characteristics of the 32 included studies.**

| **Study** | **Country** | **Centres** | **Recruit-ment** | **Valve model (type)** | **AVR n / total N** | **Age, mean ± SD (years)** | **Female %** | **Mean FU (years)** | **Target INR (aortic)** | **Follow-up completeness** |
| --- | --- | --- | --- | --- | --- | --- | --- | --- | --- | --- |
| **Van Nooten (2014)** | Belgium | Single | 1992–2011 | Medtronic/ATS Open Pivot | 1,012 / 1,382 | 61 ± 13.2 | 36.8 | 9.3 | NR | 99% |
| **Baykut (2006)** | Switzerland | Single | 1993–2003 | ATS Open Pivot | 393 / 581 | 63.7 ± 17.8 | 42.0 | 4.3 | Reduced-intensity | 99% |
| **Sezai (2010)** | Japan | Single | ~1992–2007 | ATS | 103 / 231 | 57.3 ± 11.6 | 38.1 | 6.9 | 1.6–2.0 | 99.1% |
| **Taniguchi (2012)** | Japan | Single | 1999–2010 | ATS Open Pivot | 157 / 259 | 58.8 ± 10.6 | 50.6 | 4.4 | NR | 96.6% |
| **Baudet (1995)** | France | Single | 1978–1987 | St. Jude | 773 / 1,112 | 55.9 ± 18.3 | 38.0 | 9.8 | NR | 97.5% |
| **Lund (2000)** | Denmark | Single | 1980–1993 | St. Jude standard | 694 / 694 | 58 ± 14 | 38.5 | 6.5 * | 2.0–3.0 | ~complete |
| **Emery (2005)** | USA | Single (prospective DB) | 1977–2002 | St. Jude | 1,696 / 4,480 | 64 ± 13 | 42.7 | 7.2 | 1.8–2.5 | 95% |
| **Minakata (2015)** | Japan | multicentre (5) | 1991–2001 | St. Jude | 801 / 801 | 58.3 ± 11.7 | 34.6 | 11.6 | NR | 84.1% |
| **Aoyagi (1994)** | Japan | Single | 1980–1992 | St. Jude | 178 / 908 | 52 ± 12 | 56.8 | 4.3 | NR | 94% |
| **Okamura (2020)** | Japan | Single | 2005–2011 | St. Jude 17-mm | 80 / 80 | 73 ± 7.6 | 92.5 | 8.7 | NR | 100% |
| **Johnson (2019)** | USA | Single (prospective DB) | 1979–2014 | St. Jude | 584 / 1,023 | 54 ± 14 | 42.3 | 11.0 | NR | 95% |
| **Panidis (1984)** | USA | Single | 1978–~1982 | St. Jude | 50 / 126 | 57 ± 13 | 49.2 | 2.3 | NR | echo subset |
| **Kandemir (2006)** | Turkey | Single | 1992–2004 | St. Jude | 80 / 80 | 46.4 ± 16.6 | 21.3 | 6.6 | NR | 97.7% |
| **Kim (2021)** | South Korea | Single | 1999–2015 | On-X | 344 / 861 | 51.6 ± 10.9 | 50.9 | 9.8 | NR | 93.4% |
| **Ozyurda (2005)** | Turkey | multicentre (2) | 2000–2003 | On-X | 120 / 400 | 55.6 ± 16 | 27.5 | 3.2 | NR | 99.2% |
| **Chambers (2013)** | Europe | multicentre (3) | 1998–2009 | On-X | 214 / 691 | 60.3 ± 12.6 | 41.7 | 5.2 | 2–3 | 92.4% |
| **Teshima (2017)** | Japan | Single | 2006–2014 | On-X | 78 / 78 | 65 ± 11 | 34.6 | 5.0 | 1.5–2.0 | 97.3% |
| **Moidl (2002)** | Europe/N. Am. | multicentre (20) | 1996–2001 | On-X | 303 / 532 | 59.4 ± 10 | 44.6 | 1.9 | NR | 97.2% |
| **Palatianos (2007)** | Multinational | multicentre | ~1996–2004 | On-X | 184 / 301 | 60.2 ± 8.4 | 41.9 | 4.7 | NR | 98% |
| **Chan (2010)** | Canada | multicentre | 2003–2008 | On-X | 400 / 682 | 56.9 ± 10.6 | 41.9 | 2.8 * | 2.0–2.5 | 100% |
| **Senanayake (2015)** | UK | Single (record linkage) | 1997–2010 | CarboMedics Top-Hat | 253 / 253 | 60 ± 10 | 26.1 | 8.3 | NR | ~99–100% |
| **Bouchard (2014)** | Canada | Single (prospective DB) | 1988–2010 | CarboMedics | 2,242 / 3,297 | 57 ± 11 | 41.8 | 7.5 | 2.5–3.5 | 77% |
| **Kandemir (2006)** | Turkey | Single | 1992–2004 | CarboMedics | 94 / 94 | 48.8 ± 16.1 | 23.4 | 5.8 | NR | 97.7% |
| **Kuwaki (2002)** | Japan | Single | ~1990–2000 | CarboMedics | 69 / 144 | 51.7 ± 12 | 67.4 | 6.5 | 2.0–3.0 | 95% |
| **Bernal (2005)** | Spain | Single | 1993–2001 | CarboMedics Top-Hat | 269 / 269 | 63.9 ± 13.3 | 53.2 | 6.9 | NR | 97.6% |
| **Kang (2005)** | South Korea | Single | –2001 | CarboMedics | 179 / 850 | 44.5 ± 12.5 | 53.1 | 7.9 | NR | 95.3% |
| **Tominaga (2005)** | Japan | Single | 1990–2000 | CarboMedics | 173 / 505 | 57 ± 15 | 46.9 | 5.1 | NR (Thrombotest) | 99.2% |
| **Misawa (2007)** | Japan | Single | ~1993–2005 | Sorin Bicarbon | 44 / 105 | 61.2 ± 11.3 | 47.6 | 6.1 | 1.8–3.3 | 100% |
| **Borman (1998)** | Europe | multicentre | 1990–1996 | Sorin Bicarbon | 726 / 1,351 | 58.4 ± 12.4 | 40.3 | 6.0 | NR | 98% |
| **Borman (2002)** | Europe (14) | multicentre | 1990–1996 | Sorin Bicarbon | 1,026 / 1,875 | 58 ± 12 | 40.9 | 4.6 * | NR | 98.9% |
| **Vitale (2004)** | Italy | Single | 1992–2002 | Sorin Bicarbon | 156 / 328 | 62 ± 12 | 51.2 | 3.3 | NR | 94.8% |
| **Spiliopoulos (2009)** | Germany | Single | ~1996–2006 | Sorin Bicarbon | 415 / 587 | 61.5 ± 17.4 | 37.3 | 5.8 | NR | 95.6% |

“AVR n / total N” gives the number of isolated aortic valve replacement (AVR) patients contributing to the clinical-event analysis and the total cohort of mechanical-valve recipients. Isolated-AVR counts exclude combined and concomitant-CABG procedures where the source study allowed this distinction. Age, sex and follow-up are cohort-level values as extracted from the source articles. Centres: single- vs multicentre (number of centres in parentheses). Target INR refers to isolated AVR where specified; “NR” = not reported. Follow-up completeness is the reported proportion of the cohort with complete follow-up, or the method ensuring it (registry/record linkage). Mean follow-up taken from the value explicitly reported in the source article. Late valve-related events were defined per the AATS/STS/EACTS guidelines (Edmunds 1996 / Akins 2008) in all studies except Panidis 1984 (echocardiographic/clinical assessment). The number of studies (k), patients, events and aortic patient-years contributing to each pooled outcome are reported in Tables 2–3.

**Supplementary Table 4. Risk-of-bias assessment of the included studies using the Newcastle–Ottawa Scale adapted for single-arm surgical cohorts**

| **Study** | **Selection** | | | **Outcome** | | | **Score** | **Domain-level justification** |
| --- | --- | --- | --- | --- | --- | --- | --- | --- |
|  | **Representativeness of the exposed cohort** | **Ascertainment of exposure** | **Outcome not present at start of study** | **Assessment of outcome** | **Follow-up long enough for outcomes to occur** | **Adequacy of follow-up of cohort** | **(max 6)** |  |
| **Guido J. Van Nooten, et al (2014)** | **★** | **★** | **★** | **★** | **★** | **★** | **6** | Consecutive series of all 1,382 Open Pivot recipients; exposure from secure operative records; late valve-related events absent at implantation; outcomes defined per AATS/STS guidelines with echocardiographic/registry confirmation; long-term follow-up (max 20.4 y); follow-up 99% complete (100% for residents). |
| **Doan Baykut, et al (2006)** | **★** | **★** | **★** | **★** | **★** | **★** | **6** | All consecutive ATS Open Pivot recipients; operative records; late events absent at baseline; Edmunds guideline event definitions; adequate follow-up duration (2,500 patient-years); follow-up 99% complete. |
| **Akira Sezai, et al (2010)** | **★** | **★** | **★** | **★** | **★** | **★** | **6** | Consecutive ATS recipients; secure records; late events absent at implantation; standardized AATS/STS definitions; multi-year follow-up; follow-up virtually complete (only 2 patients not traced). |
| **Shinichiro Taniguchi, et al (2012)** | **★** | **★** | **★** | **★** | **★** | **★** | **6** | Consecutive single-centre ATS series; operative records; late events absent at baseline; guideline-based (Edmunds/Akins) definitions; adequate follow-up; 96.6% complete. |
| **Eugene M. Baudet, et al (1995)** | **★** | **★** | **★** | **★** | **★** | **★** | **6** | Consecutive SJM recipients (1,112 patients / 1,244 valves); secure records; late events absent at implantation; Edmunds guideline definitions; long-term follow-up (max 15 y); 97.5% complete. |
| **Ole Lund, et al (2000)** | **★** | **★** | **★** | **★** | **★** | **★** | **6** | Consecutive standard SJM aortic recipients; Danish single centre with detailed, registry-supported follow-up (max ~19 y); late events absent at baseline; Edmunds definitions; follow-up essentially complete. |
| **Robert W. Emery, et al (2005)** | **★** | **★** | **★** | **★** | **★** | **★** | **6** | Consecutive single-valve SJM recipients (25-year experience, 32,190 patient-years); prospective database; late events absent at implantation; Edmunds definitions; long follow-up; 95% complete. |
| **Kenji Minakata, et al (2015)** | **★** | **★** | **★** | **★** | **★** | **—** | **5** | Multicentre SJM cohort; secure records/registry; late events absent at baseline; AATS/STS definitions; long follow-up (mean 11.6 y, max 23.3 y). Adequacy star not awarded: 10-year follow-up completeness only 84.1%, acknowledged by the authors as potentially influencing results. |
| **Shigeaki Aoyagi, et al (1994)** | **★** | **★** | **★** | **★** | **★** | **★** | **6** | Consecutive SJM recipients (908 patients); secure records; late events absent at implantation; Edmunds definitions; multi-year follow-up; 94% complete. |
| **Homare Okamura, et al (2020)** | **—** | **★** | **★** | **★** | **★** | **★** | **5** | Retrospective cohort with 100% complete follow-up (status confirmed >=4 y in all patients), guideline-based definitions and secure records. Representativeness star not awarded: cohort restricted to 17-mm valves only, a selected small-annulus/elderly subgroup not representative of the average mechanical AVR recipient. |
| **Scott Johnson, et al (2019)** | **★** | **★** | **★** | **★** | **★** | **★** | **6** | Prospectively followed consecutive SJM recipients (30-year experience); secure records; late events absent at baseline; AATS/STS definitions; long follow-up; 95% complete. |
| **Ioannis P. Panidis, et al (1984)** | **★** | **★** | **—** | **★** | **★** | **—** | **4** | Clinical/echocardiographic evaluation of 126 patients. Two stars not awarded: (Selection) cross-sectional echo assessment of survivors does not establish outcome-free status at a defined implantation baseline; (Outcome) 13 patients lost and follow-up limited to an echo-returning subset (mean 28 mo), so completeness of the implanted cohort cannot be demonstrated. |
| **Ozer Kandemir, et al (2006) [St. Jude]** | **★** | **★** | **★** | **★** | **★** | **★** | **6** | Consecutive SJM/CarboMedics comparative series (174 patients); secure records; late events absent at implantation; STS guideline definitions; long-term follow-up (mean 79 mo); 97.7% complete. |
| **Ji Seong Kim, et al (2021)** | **★** | **★** | **★** | **★** | **★** | **★** | **6** | Consecutive On-X recipients (up to 20 y); records plus national death database; late events absent at baseline; AATS/STS definitions; 93.4% complete (8,406 patient-years). |
| **Umit Ozyurda, et al (2005)** | **★** | **★** | **★** | **★** | **★** | **★** | **6** | Prospective consecutive On-X series (400 patients); secure records; late events absent at implantation; Edmunds definitions; 99.2% complete. |
| **John B. Chambers, et al (2013)** | **★** | **★** | **★** | **★** | **★** | **★** | **6** | Consecutive multicentre On-X cohort (follow-up to 12 y); records; late events absent at baseline; AATS/STS definitions; 92.4% complete (7.6% lost). |
| **Hideki Teshima, et al (2017)** | **★** | **★** | **★** | **★** | **★** | **★** | **6** | Consecutive On-X aortic recipients; secure records; late events absent at implantation; guideline definitions; 10-year follow-up; 97.3% complete. |
| **Reinhard Moidl, et al (2002)** | **★** | **★** | **★** | **★** | **★** | **★** | **6** | Multicentre On-X trial cohort; prospective clinic-based follow-up; late events absent at baseline; Edmunds definitions; 5-year follow-up; 97.2% complete (2.8% lost). |
| **George M. Palatianos, et al (2007)** | **★** | **★** | **★** | **★** | **★** | **★** | **6** | Multicentre On-X study (follow-up to 6.9 y); records; late events absent at implantation; AATS/STS definitions; 98% complete for AVR patients. |
| **Vincent Chan, et al (2010)** | **★** | **★** | **★** | **★** | **★** | **★** | **6** | Prospective multicentre On-X cohort; registry-based ascertainment; late events absent at baseline; AATS/STS definitions; 100% complete. |
| **Eshan L. Senanayake, et al (2015)** | **★** | **★** | **★** | **★** | **★** | **★** | **6** | CarboMedics Top-Hat cohort with a robust follow-up system; late events absent at implantation; guideline definitions; complete clinical follow-up ensured through national record linkage (Hospital Episode Statistics / ONS), ~99-100%. |
| **Denis Bouchard, et al (2014)** | **★** | **★** | **★** | **★** | **★** | **—** | **5** | Consecutive CarboMedics recipients (20-year experience); prospective database; late events absent at baseline; Edmunds definitions; long follow-up. Adequacy star not awarded: follow-up only 77% complete. |
| **Ozer Kandemir, et al (2006) [Carbomedics]** | **★** | **★** | **★** | **★** | **★** | **★** | **6** | Same comparative series as the St. Jude row (174 patients, 97.7% complete follow-up, STS definitions); identical methodology and therefore identical NOS score. |
| **Kenji Kuwaki, et al (2002)** | **★** | **★** | **★** | **★** | **★** | **★** | **6** | Consecutive CarboMedics recipients; secure records; late events absent at implantation; Edmunds definitions; 10-year follow-up; 95% complete. |
| **Jose M. Bernal, et al (2005)** | **★** | **★** | **★** | **★** | **★** | **★** | **6** | Consecutive CarboMedics Top-Hat recipients (269 patients); secure records; late events absent at baseline; guideline definitions; 10-year follow-up; 97.6% complete. |
| **Chang Hyun Kang, et al (2005)** | **★** | **★** | **★** | **★** | **★** | **★** | **6** | Consecutive CarboMedics recipients; national death-database linkage; late events absent at implantation; AATS/STS definitions; mean follow-up ~7 y; 95.3% complete. |
| **Ryuji Tominaga, et al (2005)** | **★** | **★** | **★** | **★** | **★** | **★** | **6** | Consecutive CarboMedics cohort; secure records; late events absent at baseline; Edmunds/AATS definitions; 10-year follow-up; ~99% complete. |
| **Yoshio Misawa, et al (2007)** | **★** | **★** | **★** | **★** | **★** | **★** | **6** | Consecutive Bicarbon recipients; secure records; late events absent at implantation; Edmunds definitions; multi-year follow-up; 100% complete. |
| **J.B. Borman, et al (1998)** | **★** | **★** | **★** | **★** | **★** | **★** | **6** | Bicarbon European multicentre clinical evaluation; records; late events absent at baseline; Edmunds definitions; adequate follow-up; 98% complete. |
| **J.B. Borman, et al (2002)** | **★** | **★** | **★** | **★** | **★** | **★** | **6** | Collaborative 10-year Bicarbon experience (8,280 patient-years); records; late events absent at implantation; AATS/STS definitions; 98.9% complete. |
| **Nicola Vitale, et al (2004)** | **★** | **★** | **★** | **★** | **★** | **★** | **6** | Consecutive Bicarbon recipients; secure records; late events absent at baseline; Edmunds definitions; midterm follow-up; 94.8% complete. |
| **Kyriakos Spiliopoulos, et al (2009)** | **★** | **★** | **★** | **★** | **★** | **★** | **6** | Consecutive Bicarbon recipients (614 patients); secure records; late events absent at implantation; Edmunds definitions; ~10-year follow-up; 95% complete. |

One star is awarded per item. Studies scoring 5–6 were judged at low risk of bias, 4 at moderate risk, and ≤3 at high risk.

**Supplementary Table 5. Validation of the estimated person‑time.**

| A | **Person-time set** \| **Patient-years**; Whole - reported (23) \| 133,326; Whole - estimated (same 23) \| 139,939; Whole - estimated (all 32) \| 171,645; Whole - hybrid (23 rep + 9 est) \| 165,032; AVR - reported apportioned (23) \| 71,614; AVR - estimated (same 23) \| 76,497; AVR - estimated (all 32) [primary] \| 98,987; AVR - hybrid (23 rep + 9 est) \| 94,104 | B | **Scenario** \| **All-cause mortality (95% CI)**; Primary (avr x FU) \| 2.39 (1.92-2.99); Mortality-adjusted (deaths ~ half FU) \| 2.62 (2.05-3.35); Conservative -10% person-time \| 2.66 (2.13-3.32); Conservative -20% person-time \| 2.99 (2.40-3.74) |
| --- | --- | --- | --- |
| C |  | D |  |

Person‑time was estimated as number of patients × mean follow‑up. Because clinical events were available only for the isolated‑aortic (AVR) subgroup, the figure compares person‑time itself (rates only in panel B).

(A) Total person‑time for the whole population and the AVR subgroup under four definitions: reported by the studies (23 studies), estimated for those same 23, estimated for all 32, and a hybrid (reported where available, estimated for the rest).

(B) Pooled all‑cause mortality rate (per 100 patient‑years; 95% CI) under the primary person‑time and three progressively conservative reductions of it (mortality‑adjusted; −10%; −20%).

(C, D) Reported versus estimated person‑time in the 23 studies that reported it, for the whole population (C) and the apportioned aortic subgroup (D); each point is a study, the dashed line is identity and the red line the fitted regression.

AVR, isolated aortic valve replacement; CI, confidence interval; FU, follow‑up; r, Pearson correlation; n, number of studies.

**Supplementary Table 6. Univariable meta-regression — all-cause mortality and reintervention**

| **Outcome** | **Moderator** | **k** | **beta** | **CI95** | **P** | **R2** | **tau2_res** |
| --- | --- | --- | --- | --- | --- | --- | --- |
| All-cause mortality | Publication year | 31 | -0.0149 | -0.0447, 0.0150 | 0.317 | 0.1 | 0.314 |
| All-cause mortality | Sample size (n) | 31 | 0.0002 | -0.0000, 0.0004 | 0.087 | 7.6 | 0.322 |
| All-cause mortality | Mean age (years) | 31 | 0.0390 | 0.0016, 0.0763 | 0.041 | 12.1 | 0.286 |
| All-cause mortality | Female (%) | 31 | 0.0141 | -0.0026, 0.0307 | 0.095 | 7.1 | 0.305 |
| Reintervention | Publication year | 25 | -0.0228 | -0.0604, 0.0148 | 0.222 | 5.0 | 0.245 |
| Reintervention | Sample size (n) | 25 | -0.0002 | -0.0004, 0.0001 | 0.128 | 12.4 | 0.187 |
| Reintervention | Mean age (years) | 25 | 0.0343 | -0.0311, 0.0996 | 0.290 | 1.6 | 0.226 |
| Reintervention | Female (%) | 25 | 0.0299 | -0.0009, 0.0607 | 0.056 | 23.4 | 0.147 |

Moderator=study-level covariate tested; k=number of studies in the model; Coef=meta-regression slope (change in the log incidence rate per one-unit increase in the moderator); CI=95% confidence interval of the slope; p=significance of the slope (Hartung-Knapp); R2=proportion of between-study heterogeneity explained by the moderator; tau2_res= residual between-study variance after adjustment.

**Supplementary Table 7. Pairwise comparisons between valve models.**

| **All-cause mortality** |  |  |  | |  |
| --- | --- | --- | --- | --- | --- |
| **Comparison** | **Rate ratio (95% CI)** | **p** | **k (A vs B)** | |  |
| Bicarbon vs Carbomedics | 0.79 (0.46-1.35) | 0.353 | 4 vs 7 | |  |
| Bicarbon vs Medtronic/ATS | 1.24 (0.65-2.37) | 0.438 | 4 vs 4 | |  |
| Bicarbon vs On-X | 0.86 (0.38-1.96) | 0.685 | 4 vs 7 | |  |
| Bicarbon vs St. Jude | 1.40 (0.59-3.31) | 0.408 | 4 vs 9 | |  |
| Carbomedics vs Medtronic/ATS | 1.58 (0.88-2.82) | 0.112 | 7 vs 4 | |  |
| Carbomedics vs On-X | 1.14 (0.59-2.21) | 0.664 | 7 vs 7 | |  |
| Carbomedics vs St. Jude | 1.88 (0.96-3.68) | 0.063 | 7 vs 9 | |  |
| Medtronic/ATS vs On-X | 0.72 (0.31-1.69) | 0.406 | 4 vs 7 | |  |
| Medtronic/ATS vs St. Jude | 1.19 (0.50-2.85) | 0.670 | 4 vs 9 | |  |
| On-X vs St. Jude | 1.67 (0.76-3.66) | 0.182 | 7 vs 9 | |  |
| **Valve-related mortality** |  |  | |  | |
| **Comparison** | **Rate ratio (95% CI)** | **p** | | **k (A vs B)** | |
| Bicarbon vs Carbomedics | 0.77 (0.43-1.37) | 0.326 | | 5 vs 5 | |
| Bicarbon vs Medtronic/ATS | 0.38 (0.13-1.10) | 0.067 | | 5 vs 2 | |
| Bicarbon vs On-X | 0.32 (0.15-0.68) | 0.009 * | | 5 vs 4 | |
| Bicarbon vs St. Jude | 1.65 (0.85-3.20) | 0.126 | | 5 vs 8 | |
| Carbomedics vs Medtronic/ATS | 0.50 (0.13-1.97) | 0.250 | | 5 vs 2 | |
| Carbomedics vs On-X | 0.43 (0.15-1.20) | 0.093 | | 5 vs 4 | |
| Carbomedics vs St. Jude | 2.09 (0.99-4.40) | 0.052 | | 5 vs 8 | |
| Medtronic/ATS vs On-X | 0.85 (0.43-1.68) | 0.540 | | 2 vs 4 | |
| Medtronic/ATS vs St. Jude | 4.07 (1.03-16.13) | 0.047 * | | 2 vs 8 | |
| On-X vs St. Jude | 4.99 (1.70-14.65) | 0.008 * | | 4 vs 8 | |
| **Reintervention** | | | | |  |
| **Comparison** | **Rate ratio (95% CI)** | **p** | **k (A vs B)** | |  |
| Bicarbon vs Carbomedics | 0.85 (0.34-2.15) | 0.703 | 5 vs 7 | |  |
| Bicarbon vs Medtronic/ATS | NE | - | 5 vs 0 | |  |
| Bicarbon vs On-X | 0.70 (0.24-2.07) | 0.480 | 5 vs 6 | |  |
| Bicarbon vs St. Jude | 0.77 (0.40-1.50) | 0.404 | 5 vs 7 | |  |
| Carbomedics vs Medtronic/ATS | NE | - | 7 vs 0 | |  |
| Carbomedics vs On-X | 0.84 (0.26-2.71) | 0.754 | 7 vs 6 | |  |
| Carbomedics vs St. Jude | 0.89 (0.40-1.97) | 0.756 | 7 vs 7 | |  |
| Medtronic/ATS vs On-X | NE | - | 0 vs 6 | |  |
| Medtronic/ATS vs St. Jude | NE | - | 0 vs 7 | |  |
| On-X vs St. Jude | 1.07 (0.42-2.73) | 0.869 | 6 vs 7 | |  |
| **Bleeding** | | | | |  |
| **Comparison** | **Rate ratio (95% CI)** | **p** | **k (A vs B)** | |  |
| Bicarbon vs Carbomedics | 1.01 (0.52-1.94) | 0.975 | 5 vs 6 | |  |
| Bicarbon vs Medtronic/ATS | 0.65 (0.25-1.64) | 0.294 | 5 vs 3 | |  |
| Bicarbon vs On-X | 0.98 (0.41-2.31) | 0.954 | 5 vs 7 | |  |
| Bicarbon vs St. Jude | 1.62 (0.66-3.99) | 0.269 | 5 vs 9 | |  |
| Carbomedics vs Medtronic/ATS | 0.64 (0.25-1.65) | 0.301 | 6 vs 3 | |  |
| Carbomedics vs On-X | 0.98 (0.44-2.17) | 0.951 | 6 vs 7 | |  |
| Carbomedics vs St. Jude | 1.63 (0.72-3.67) | 0.219 | 6 vs 9 | |  |
| Medtronic/ATS vs On-X | 1.50 (0.46-4.88) | 0.450 | 3 vs 7 | |  |
| Medtronic/ATS vs St. Jude | 2.46 (0.73-8.29) | 0.130 | 3 vs 9 | |  |
| On-X vs St. Jude | 1.67 (0.69-4.08) | 0.235 | 7 vs 9 | |  |
| **Thromboembolic events** | | | | |  |
| **Comparison** | **Rate ratio (95% CI)** | **p** | **k (A vs B)** | |  |
| Bicarbon vs Carbomedics | 1.22 (0.60-2.46) | 0.537 | 5 vs 6 | |  |
| Bicarbon vs Medtronic/ATS | 1.30 (0.46-3.69) | 0.563 | 5 vs 3 | |  |
| Bicarbon vs On-X | 1.24 (0.75-2.06) | 0.365 | 5 vs 7 | |  |
| Bicarbon vs St. Jude | 1.66 (0.85-3.25) | 0.127 | 5 vs 9 | |  |
| Carbomedics vs Medtronic/ATS | 1.00 (0.33-3.05) | 0.993 | 6 vs 3 | |  |
| Carbomedics vs On-X | 1.06 (0.58-1.92) | 0.838 | 6 vs 7 | |  |
| Carbomedics vs St. Jude | 1.32 (0.69-2.53) | 0.366 | 6 vs 9 | |  |
| Medtronic/ATS vs On-X | 0.93 (0.43-2.01) | 0.838 | 3 vs 7 | |  |
| Medtronic/ATS vs St. Jude | 1.31 (0.47-3.60) | 0.568 | 3 vs 9 | |  |
| On-X vs St. Jude | 1.31 (0.73-2.33) | 0.342 | 7 vs 9 | |  |

Random-effects meta-regression restricted to each pair of valve models on the log incidence-rate scale (Empirical-Bayes tau2, Hartung-Knapp CIs). Rate ratio (RR) is the second model versus the first; RR>1 indicates a higher rate in the second model. k = contributing studies per model; NE = not estimable (<2 studies in a model). * p<0.05.

**Supplementary Table 8. Heterogeneity and small-study effects, per mortality outcome**

| **Outcome** | **k** | **I2** | **tau2** | **Egger_p** | **TrimFill** |
| --- | --- | --- | --- | --- | --- |
| All-cause mortality | 31 | 96% | 0.3129 | 0.002 | 8 |
| Valve-related mortality | 24 | 86% | 0.2971 | 0.018 | 8 |
| - Thrombosis (death) | 17 | 0% | 0.0000 | 0.737 | 2 |
| - Thromboembolism (death) | 17 | 47% | 0.1470 | 0.018 | - |
| - Bleeding (death) | 19 | 55% | 0.1595 | 0.007 | 9 |
| - Endocarditis (death) | 17 | 15% | 0.0594 | 0.047 | 7 |
| - Paravalvular leak (death) | 14 | 0% | 0.0000 | 0.176 | 3 |
| - Reoperation (death) | 11 | 0% | 0.0000 | 0.531 | 2 |
| - Others (death) | 13 | 89% | 2.1060 | 0.013 | 6 |

Column key: k = contributing studies; I2 and tau2 summarise between-study heterogeneity; Egger's p tests funnel-plot asymmetry and is reported when >=10 studies are available; trim-and-fill gives the number of studies imputed under a symmetry assumption (0 = symmetric funnel).

**Supplementary Table 9. Heterogeneity and small-study effects, per event outcome**

| **Outcome** | **k** | **I2** | **tau2** | **Egger_p** | **TrimFill** |
| --- | --- | --- | --- | --- | --- |
| Thromboembolic events | 30 | 84% | 0.1780 | 0.001 | 3 |
| Valve thrombosis | 27 | 22% | 0.1145 | 0.014 | - |
| Pannus | 12 | 36% | 0.1607 | 0.006 | 6 |
| Paravalvular leak | 21 | 48% | 0.2259 | 0.256 | 6 |
| Endocarditis | 27 | 58% | 0.1904 | 0.128 | 8 |
| Bleeding | 30 | 92% | 0.3389 | 0.000 | 9 |
| Reintervention (total) | 25 | 68% | 0.2242 | 0.343 | 7 |
| - reint: PVL | 15 | 0% | 0.0000 | 0.861 | 2 |
| - reint: Endocarditis | 16 | 60% | 0.4013 | 0.457 | 2 |
| - reint: Thrombosis | 13 | 0% | 0.0000 | 0.957 | 0 |
| - reint: Pannus | 12 | 43% | 0.5446 | 0.009 | 3 |
| - reint: Others | 13 | 25% | 0.1477 | 0.012 | 6 |

Column key: k = contributing studies; I2 and tau2 summarise between-study heterogeneity;Egger's p tests funnel-plot asymmetry and is reported when >=10 studies are available; trim-and-fill gives the number of studies imputed under a symmetry assumption (0 = symmetric funnel).

**Supplementary Table 10. Leave-one-out — all-cause mortality**

| **Study excluded (valve model)** | **Rate for the valve group** | **Rate overall** |
| --- | --- | --- |
| J.B, Borman (1998) - Bicarbon | 2.20 | 2.39 |
| J.B, Borman* (2002) - Bicarbon | 2.31 | 2.40 |
| Nicola Vitale (2004) - Bicarbon | 2.24 | 2.39 |
| Yoshio Misawa (2007) - Bicarbon | 2.04 | 2.36 |
| Kyriakos Spiliopoulos (2009) - Bicarbon | 2.40 | 2.42 |
| Kenji Kuwaki (2002) - Carbomedics | 1.74 | 2.40 |
| Jose M, Bernal (2005) - Carbomedics | 1.66 | 2.39 |
| Chang Hyun Kang (2005) - Carbomedics | 1.86 | 2.44 |
| Ryuji Tominaga (2005) - Carbomedics | 1.68 | 2.39 |
| Ozer Kandemir (2006) - Carbomedics | 1.81 | 2.43 |
| Denis Bouchard (2014) - Carbomedics | 1.68 | 2.40 |
| Eshan L Senanayake (2015) - Carbomedics | 2.11 | 2.49 |
| Doan Baykut (2006) - Medtronic/ATS | 2.32 | 2.34 |
| Akira Sezai (2010) - Medtronic/ATS | 3.15 | 2.42 |
| Shinichiro Taniguchi (2012) - Medtronic/ATS | 2.69 | 2.37 |
| Guido J. Van Nooten (2014) - Medtronic/ATS | 3.00 | 2.40 |
| Reinhard Moidl (2002) - On-X | 2.05 | 2.43 |
| Ümit Özyurda (2005) - On-X | 2.16 | 2.45 |
| George M, Palatianos (2007) - On-X | 1.91 | 2.40 |
| Vincent Chan (2010) - On-X | 1.79 | 2.37 |
| John B, Chambers (2013) - On-X | 2.23 | 2.47 |
| Hideki Teshima (2017) - On-X | 1.66 | 2.32 |
| Ji Seong Kim (2021) - On-X | 1.98 | 2.42 |
| Ioannis P Panidis (1984) - St. Jude | 3.37 | 2.40 |
| Shigeaki Aoyagi (1994) - St. Jude | 2.84 | 2.29 |
| Euge M, Baudet (1995) - St. Jude | 3.44 | 2.40 |
| Ole Lund (2000) - St. Jude | 3.11 | 2.34 |
| Robert W. Emery (2005) - St. Jude | 2.83 | 2.29 |
| Ozer Kandemir (2006) - St. Jude | 3.52 | 2.42 |
| Kenji Minakata (2015) - St. Jude | 3.30 | 2.38 |
| Scott Johnson (2019) - St. Jude | 3.71 | 2.45 |
| Homare Okamura (2020) - St. Jude | 3.15 | 2.35 |

Each row re-estimates the pooled all-cause mortality rate after omitting a single study, reported both for that study's valve-model subgroup ("Rate for the valve group") and for the whole analysis ("Rate overall"). Rates are expressed per 100 patient-years and were pooled with a random-effects Poisson model on the log incidence-rate scale (Empirical Bayes/Paule-Mandel τ², Hartung-Knapp confidence intervals) on the isolated-aortic person-time denominator.

**Supplementary Figure 1. Contour-enhanced funnel plots for the principal outcomes.**

Contour-enhanced funnel plots of the pooled incidence rates for all-cause mortality, valve-related mortality, reintervention, thromboembolic events and bleeding. Each point represents one study; the horizontal axis shows the log incidence rate and the vertical axis the standard error (more precise studies, with smaller standard error, lie towards the top). The vertical line is the random-effects pooled log-rate, and the nested shaded regions are its 90%, 95% and 99% pseudo-confidence contours; in the absence of small-study effects and heterogeneity, studies would scatter symmetrically within the inverted funnel. Small-study effects were formally tested with Egger's regression and trim-and-fill (Supplementary Tables 8 and 9); rates are per 100 patient-years, pooled with a random-effects Poisson model on the log incidence-rate scale (Empirical Bayes/Paule–Mandel τ², Hartung–Knapp confidence intervals). Funnel-based methods have limited validity under the strong heterogeneity of incidence-rate meta-analyses and are therefore interpreted cautiously.
